# A Common Function of Basal Ganglia-Cortical Circuits Subserving Speed in Both Motor and Cognitive Domains

**DOI:** 10.1523/ENEURO.0200-17.2017

**Published:** 2017-12-08

**Authors:** Takashi Hanakawa, Andrew M. Goldfine, Mark Hallett

**Affiliations:** 1 Department of Advanced Neuroimaging, Integrative Brain Imaging Center, National Center of Neurology and Psychiatry, Kodaira, Tokyo 187-8551, Japan; 2 Human Motor Control Section, National Institute of Neurological Disorders and Stroke, National Institutes of Health, Bethesda, MD 20892; 3 Department of Neurology, Stony Brook School of Medicine, Stony Brook, NY 11794

**Keywords:** cognitive effort, dopamine, frontal cortex, motor imagery

## Abstract

Distinct regions of the frontal cortex connect with their basal ganglia and thalamic counterparts, constituting largely segregated basal ganglia-thalamo-cortical (BTC) circuits. However, any common role of the BTC circuits in different behavioral domains remains unclear. Indeed, whether dysfunctional motor and cognitive BTC circuits are responsible for motor slowing and cognitive slowing, respectively, in Parkinson’s disease (PD) is a matter of debate. Here, we used an effortful behavioral paradigm in which the effects of task rate on accuracy were tested in movement, imagery, and calculation tasks in humans. Using nonlinear fitting, we separated baseline accuracy (*A_base_*) and “agility” (ability to function quickly) components of performance in healthy participants and then confirmed reduced agility and preserved *A_base_* for the three tasks in PD. Using functional magnetic resonance imaging (fMRI) and diffusion tractography, we explored the neural substrates underlying speeded performance of the three tasks in healthy participants, suggesting the involvement of distinct BTC circuits in cognitive and motor agility. Language and motor BTC circuits were specifically active during speeded performance of the calculation and movement tasks, respectively, whereas premotor BTC circuits revealed activity for speeded performance of all tasks. Finally, PD showed reduced task rate-correlated activity in the language BTC circuits for speeded calculation, in the premotor BTC circuit for speeded imagery, and in the motor BTC circuits for speeded movement, as compared with controls. The present study casts light on the anatomo-functional organization of the BTC circuits and their parallel roles in invigorating movement and cognition through a function of dopamine.

## Significance Statement

The frontal cortex, basal ganglia and thalamus constitute largely segregated multiple basal ganglia-thalamo-cortical (BTC) circuits. However, any common role of the BTC circuits in different behavioral domains remains unclear. Using a novel behavioral paradigm, we identified distinct BTC circuits as the neural substrates of speeded performance in calculation, imagery and movement tasks in healthy humans. We also found dysfunctions of the same BTC circuits were responsible for slowed performance in participants with Parkinson’s disease (PD). The present study indicates a function spanning multiple BTC circuits to invigorate both motor and cognitive frontal regions, thereby allowing for enhanced performance speed regardless of the task domains.

## Introduction

Limbic, cognitive, and motor regions of the frontal cortex topographically project to specific regions of the striatum. This topographical organization is maintained within the basal ganglia and the thalamus, projecting back to the frontal regions of origin and forming the parallel-basal ganglia-thalamo-cortical (BTC) circuits (Alexander et al., 1986; Middleton and Strick, 2000; Kim and Hikosaka, 2015; Haber, 2016). Thus, although updated anatomic evidence indicates substantial overlap and convergence across the BTC circuits (Averbeck et al., 2014), the central axes of the BTC circuits are topographically segregated (Kim and Hikosaka, 2015). An example of functional segregation is the somatotopic organization that is preserved throughout the BTC circuits (Nambu, 2011). Human neuroimaging studies have documented the topographical organization of the striatum, using coactivation patterns derived from a database of task functional magnetic resonance imaging (fMRI; Pauli et al., 2016), resting-state fMRI (Choi et al., 2012), and diffusion tractography (Lehericy et al., 2004a; Tziortzi et al., 2013). However, it remains unclear how differently or similarly information is processed in the multiple BTC circuits for distinct behaviors spanning from emotion, cognition to movement.

A specific type of cognitive impairment in Parkinson’s disease (PD) hints at the roles of the BTC circuits for both movement and cognition. PD is a basal ganglia disorder that is characterized by motor slowing (bradykinesia) but also by cognitive impairment even at an early stage of the disease (Kehagia et al., 2010). Among several types of cognitive disturbance, cognitive slowing (bradyphrenia) is conceptually parallel to motor slowing. Interestingly, some studies report cognitive slowing in PD (Wilson et al., 1980; Pillon et al., 1989; Cooper et al., 1994; Sawamoto et al., 2002; Lee et al., 2003; Muslimovic et al., 2005; Jokinen et al., 2013; Vlagsma et al., 2016), but some studies do not (Helscher and Pinter, 1993; Duncombe et al., 1994; Howard et al., 1994). This discrepancy results, at least in part, from the differences in paradigm designs. Most studies measure reaction times, which are influenced by movement speed and are suggested to be an unreliable measure in PD (Sawamoto et al., 2002; Ebaid et al., 2017). A way to deal with the confounding effect of movement speed is to vary the required rate of cognitive operations, and then have the motor output be an accuracy measure. When various task rates are examined, accuracy declines for high task-rate trials with limited times available for cognitive processing (i.e., rate-accuracy trade-off). This strategy was previously applied to the measurement of cognitive slowing in PD (Sawamoto et al., 2002), but the analysis did not allow them to assess cognitive speed at an individual level.

Along with the methodology for measuring cognitive speed, the understanding of the pathophysiological mechanisms responsible for cognitive slowing needs to be advanced. In contrast to the many studies that have associated motor slowing with dysfunctions of the motor BTC circuit (Playford et al., 1992; Herz et al., 2014; Michely et al., 2015), only a few neuroimaging studies have attempted to uncover the neural correlates of cognitive slowing (Sawamoto et al., 2007; Jokinen et al., 2013). Considering the conceptual parallelism of motor slowing and cognitive slowing, it is tempting to assume that dysfunctions of the cognitive BTC circuit underlie cognitive slowing similar to the dysfunctions of the motor BTC circuit underlying motor slowing. However, to our knowledge, no study has explicitly tested this assumption. Moreover, if dysfunctions of distinct BTC circuits correlate with motor and cognitive slowing, then the corresponding BTC circuits should be recruited for speeded motor and cognitive processing in a healthy population. However, the effects of a task rate on BTC activity are poorly understood. Thus, this question needs to be addressed before discussing the mechanisms of cognitive slowing.

The purposes of this study were: (1) to confirm the coexistence of motor and cognitive slowing in PD, (2) to identify the neural architecture subserving speeded motor and cognitive processing, and (3) to understand the relationship of the identified neural architecture with motor/cognitive slowing in PD. Here, we used a behavioral paradigm in which the effects of a task rate on accuracy were tested in the movement, imagery and calculation tasks. We applied this paradigm to healthy participants and participants with PD. We then conducted fMRI in healthy participants to find the substrates of speeded motor and cognitive processing, and analyzed diffusion tractography to define the BTC circuits. Finally, we conducted an fMRI experiment in participants with PD to test if dysfunctions of the identified BTC circuits were associated with motor and cognitive slowing.

## Materials and Methods

### Participants

Participants in the behavioral experiment were 46 healthy volunteers [20 females; 44.1 ± 18.2 years (mean ± SD); range, 21–77 years] and 19 volunteers with mild to moderate PD (seven females; 64.2 ± 9.4 years; range, 43–79 years). All participants were right handed and had a mini-mental state examination (MMSE) score of ≥28. Among those participants, 38 healthy volunteers (17 females; 45.7 ± 18.2 years; range, 21-77 years) and 15 volunteers with PD (six females; 63.7 ± 10.3 years; range, 43-79 years) also agreed to participate in the fMRI experiment within one week after the behavioral experiment. Profiles of the participants, including the clinical status of the participants with PD are described in Table 1 and Extended Data Table 1-1.

**Table 1.** Demographic profiles of participants: behavioral experiment

	Healthy adults (*n* = 46)			
	Nonsenior (*n* = 24)	Senior control for PD (*n* = 22)	PD (*n* = 19)	Statistics (senior control vs Parkinson)
Age (mean ± SD, y)	28.6 ± 8.3	61.1 ± 7.9	64.2 ± 9.4	*T*_(39)_ = 1.1, *p* = 0.25^†^
Male:female	15:9	11:11	12:7	χ^2^ = 1.43, *p* = 0.70^‡^
EHI (mean ± SD)	0.9 ± 0.1	0.93 ± 0.14	0.94 ± 0.19	*T*_(39)_ = 0.36, *p* = 0.83^†^
Education (mean ± SD, y)	16.3 ± 1.9	15.9 ± 3.1	17.6 ± 3.1	*T*_(39)_ = 1.7, *p* = 0.09^†^
MMSE (mean ± SD)	29.7 ± 0.6	29.5 ± 0.7	29.4 ± 0.8	*T*_(39)_ = 0.14, *p* = 0.89^†^
Experience	N = 17	N = 10	N = 10	χ^2^ = 0.46, *p* = 0.93^‡^
Hoehn-Yahr scale (mean ± SD)	NA	NA	2.0 ± 0.5	NA
UPDRS motor/bradykinesia subscale (off-medication; mean ± SD)	NA	NA	15.7 ± 8.5/6.6 ± 4.0	NA
LDE (mean ± SD)	NA	NA	534 ± 271	NA

EHI, Edinburgh Handedness Inventory; “Experience” refers to personal history that may influence finger dexterity, such as typing, piano playing, and so forth. Participants self-reported their experience in those activities in a simple yes-no questionnaire. Levodopa dose equivalency (LDE) is reported as a summary measure of anti-Parkinsonian medication. †not significant with a two-sample *t* test; ‡not significant with a χ^2^ test. NA: not applicable.

10.1523/ENEURO.0200-17.2017.t1-1 Extended Data Table 1-1 Full profiles of the subgroups of healthy participants and participants with Parkinson's disease (PD) in the fMRI experiment. Download Table 1-1, DOCX file.

For the comparison with the PD group, a subset of the healthy participants served a matched control group: 22 participants (11 females; 61.3 ± 7.7 years; range, 50-77 years) for the behavioral experiment and 18 participants (eight females; 61.5 ± 8.5 years; range, 51-77 years) for the fMRI experiment. There were no differences in age, sex, handedness, education, MMSE, or experiences concerning finger dexterity between the control participants and the participants with PD in either the behavioral experiment (Table 1) or the fMRI experiment (Extended Data Table 1-1). All participants were registered volunteers who had undergone full neurologic examination and anatomic MRI reviewed by neuroradiologists for eligibility, and gave prior informed consent approved by the institutional review board at the National Institutes of Health. Before the experiments, all participants abstained from alcohol more than 24 h. All participants with PD regularly took anti-Parkinsonian medications: levodopa-carbidopa only (*n* = 8), dopamine agonist or monoamine oxidase (MAO) inhibitor only (*n* = 4), levodopa-carbidopa plus dopamine agonist/MAO inhibitor (*n* = 7). The levodopa equivalent does of those medications is reported in Table 1. The participants with PD were off anti-Parkinsonian medication more than 12 h (practical off-state) before the experiments; thus, it is likely that the participants with PD were in a relatively low dopamine state during the experiments.

We also recruited an independent group of 15 non-senior healthy volunteers (five females; 26.7 ± 10.1 years; range, 22-43 years) who participated only in a diffusion tractography experiment to delineate the BTC circuits. All the participants were without a history of previous neuro-psychiatric disorders, and gave prior informed consent approved by the institutional review board at the National Center of Neurology and Psychiatry, Japan.

### Rate-accuracy trade-off paradigm for motor and cognitive tasks

**Figure 1.**
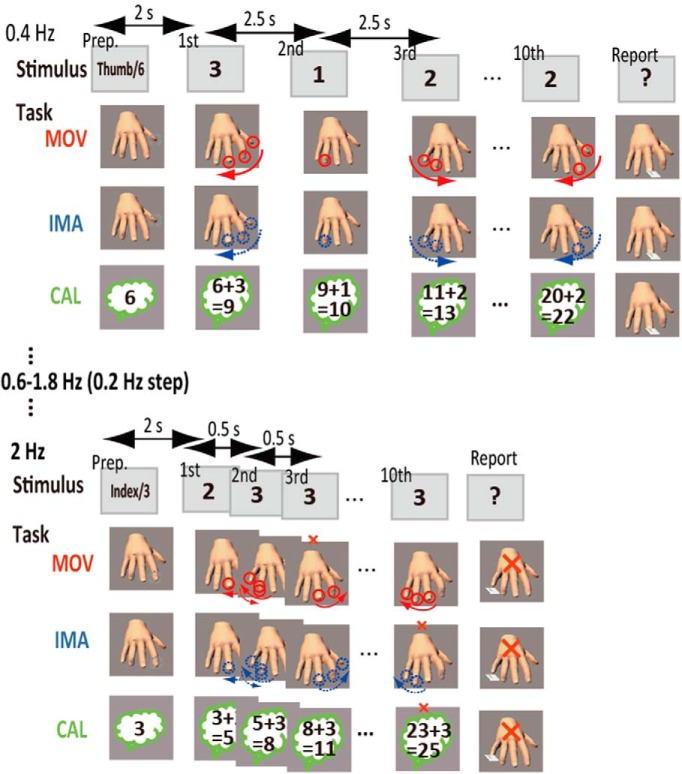
Behavioral paradigm. Participants performed movement (MOV), imagery (IMA), and calculation (CAL) tasks, all of which were guided by sets of 10 number stimuli. In MOV and IMA, after a preparatory word stimulus (Prep.) specifying a starting digit (e.g., thumb), a participant sequentially tapped their right-hand fingers according the number stimuli that specified how many taps should be made physically (solid circles) for MOV or virtually (broken circles) for IMA. Participants reported the next finger they would physically (MOV) or virtually (IMA) tap at the end of each trial. In CAL, starting from a single-digit number (e.g., 6), a participant mentally added all the presented numbers and reported the last digit of the sum. In all tasks, a single misprocessing of any one of the stimuli (a small red x-mark) should result in an erroneous response (a large red x-mark). The stimulus rate of the number stimuli was varied across trials (0.4-2 Hz with a 0.2-Hz step) so that faster processing was imposed in trials with higher rates than in those with lower rates.

Participants performed movement, imagery, and calculation tasks while sitting comfortably on a chair. The stimulus presentation and response acquisition were controlled on a personal computer using Presentation (http://www.neurobs.com/). All the tasks were initiated by a 2-s visual presentation of a preparatory stimulus, followed by a series of 10 number stimuli semi-randomly selected from 1 to 3, with each stimulus presented for 250 ms. The design of the movement and imagery tasks followed previous literature (Hanakawa et al., 2003). For the movement task, starting from a finger cued by a preparatory word stimulus (e.g., thumb), participants physically and sequentially tapped their right-hand fingers according to a semi-random sequence of number stimuli. The presented number informed a participant of how many taps to perform at each stimulus presentation (Fig. 1). Participants were asked to move their fingers as far and as fast as possible. When tapping reached the little finger or the thumb, the tapping was looped back in the opposite direction and continued. A response prompt was presented after the 10th stimulus in each trial, and participants reported the next finger they would tap, using a keypad under the right hand. For the imagery task, the participants performed the same task without muscle contraction, using a first-person perspective, visuokinesthetic, preparatory-stage, and explicit motor imagery (Hanakawa, 2016). In response to a prompt, participants reported the next finger they would virtually tap. When the same set of the stimuli was presented, the same finger was the correct answer across the movement and imagery tasks, allowing us to measure the performance of both motor execution and imagery. For the calculation task (Fig. 1), the preparatory stimulus was a semi-randomly selected single-digit number, and participants mentally added all the presented numbers (i.e., serial mental addition). Participants reported the first digit of the sum, using button press with the thumb-little finger representing both 1-5 and 6-10. Thus, the chance level was adjusted to be the same (0.2) across the tasks, allowing us to assess accuracy fairly across different task domains. Across the three tasks, the next trial did not begin until a participant made a response (no time constraint to the response). Hence, slowing of motor responses at the end of trials should not affect accuracy. In sum, all tasks placed an emphasis on robust execution of stimulus-response/operation linkage based on the same set of stimuli in each behavioral domain toward a final response. No feedback was given after the response.

To assess the rate-accuracy trade-off relationship in the three tasks, we used the following different frequencies (0.2-Hz step) for the number stimulus presentation in different trials: 0.4-2.0 Hz for all healthy participants (with additional 0.2-Hz trials for the control participants for PD) and 0.2-1.8 Hz for participants with PD. We selected these ranges of task rates according to a preliminary experiment and literature (Sawamoto et al., 2002), and the range was adjusted for a wide range of participants for efficiency. The rate was increased and then decreased in a task run, thereby including two trials for each rate. Six runs were prescribed for each task (12 trials for each rate), and the total number of trials was 324 (12 trials, nine rates, and three tasks). The participants were encouraged to take a short break between the runs. Since we failed to find the effects of acceleration or deceleration or the runs on accuracy, we pooled the data across these factors.

### Assessment of accuracy data in the behavioral experiment

In the behavioral experiment, we assessed the effects of a task rate on accuracy in the three tasks, and compared those effects between participants with PD and the matched controls. We ran two complementary analyses: an analysis of accuracy data averaged across participants for each rate and an analysis of parameters derived from a fitting analysis in each individual.

**Figure 2.**
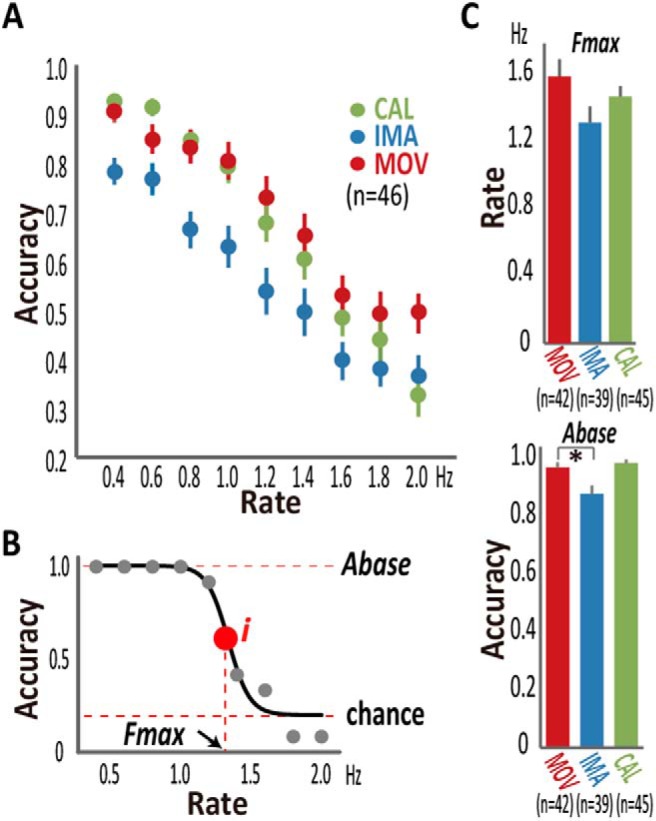
Behavioral results in healthy participants. ***A***, Accuracy, when averaged across healthy participants (*n* = 46), monotonically declined in all movement (MOV), imagery (IMA), and calculation (CAL) tasks as the task rate increased. ***B***, Sigmoid fitting (solid line) of raw (filled gray circles) accuracy data yielded the base-accuracy (*A_base_*) and agility (*F_max_*) parameters for the calculation task from a representative healthy participant. The inflection point (*i*) corresponded to the *F_max_* at which accuracy fell to the midpoint between *A_base_* and *A_chance_* (0.2). Data from some participants had to be excluded from the fitting analysis due to low accuracy and other reasons (Extended Data Fig. 2-1). ***C****, F_max_* did not significantly differ between the tasks, but *A_base_* of IMA was lower than that of MOV and marginally lower than that of CAL. Error bars: SEM, *p < 0.0.5.

The raw accuracy data averaged across participants for each rate showed a monotonic decline in accuracy as a function of the task rate for all three tasks (Fig. 2*A*). Group-averaged accuracy data were compared across the participants with PD and the controls, using repeated-measures ANOVA (RM-ANOVA) with GROUP (participants with PD and controls) as a between-subject factor and the rate and task as within-subject factors. We were especially interested in the GROUP-by-rate interactions, which reflected the differences in the effects of task rates on accuracy between the groups. While the main effects of the GROUP should reflect differences in accuracy between the groups overall, GROUP-by-rate interactions can be taken as evidence for changes in performance speed. A threshold for significance was set at *p* < 0.05 after Greenhouse-Geisser correction for sphericity as indicated by the reported degree of freedom when applicable. However, we chose not to use these results from the group-averaged accuracy data as the main outcomes of the behavioral experiment because of multiple factors affecting accuracy, each of which substantially differed across the tasks and individuals. This analysis was performed before and after the exclusion of the data according to the accuracy criteria for the fitting analysis.

10.1523/ENEURO.0200-17.2017.f2-1 Extended Data Figure 2-1 Design of the experiment and data available for each analysis step. Forty-six healthy volunteers including 24 non-seniors and 22 seniors and 19 volunteers with mild to moderate PD participated in the behavioral experiment. Among those participants, 38 healthy volunteers (20 non-seniors and 18 seniors) and 15 volunteers with PD also participated in the fMRI experiment within one week after the behavioral experiment. After the fitting analysis of accuracy data in the behavioral experiment, *A_base_* and *F_max_* parameters were available from 42 healthy (22 non-seniors and 20 senior) and 17 PD participants for the movement (MOV) task, 39 healthy (21 non-seniors and 18 seniors), and 15 PD participants for the imagery (IMA) task, and 45 healthy (23 non-seniors and 22 seniors) and 19 PD participants for the calculation (CAL) task. In the fMRI analysis, simultaneous EMG data were available from 30 healthy (14 non-seniors and 16 seniors) and 15 PD participants. We also ran a diffusion tractography experiment for diffusion-based subcortical classification in an independent group of 15 healthy non-senior participants. Download Figure 2-1, TIF file.

At the individual level, accuracy typically showed nonlinear declines across task rates, with substantial differences in the inflection point across the tasks and individuals (Fig. 2*B*). To capture the task rate-accuracy trade-off relationship individually, we used a fitting analysis with a sigmoid (Boltzmann) function (Origin Pro; OriginLab).

$$y=\frac{A_{base}–A_{chance}}{1+e^{(x-F_{max})/dx}}+A_{chance}$$

where *y* denotes accuracy, *x* task rate, *A_base_* baseline accuracy, *A_chance_* chance level, *F_max_* the estimation of the rate (Hz) at which accuracy decreased to the halfway point between *A_base_* and *A_chance_*, and *dx* time constant. *A_chance_* had a fixed value of 0.2 (chance level), but the other parameters were initially unfixed. The fitting reasonably explained the rate-accuracy trade-off relationship across the three tasks at an individual level. *A_base,_* ranging from 0 to 1, corresponded to the accuracy when time constraints were minimal. When the initial fitting reported *A_base_* > 1, fitting was redone with a fixed *A_base_* value of 1. *F_max_* represents ability to function quickly (defined as agility), indexing how fast a participant performed the task with time constraints while maintaining reasonable accuracy. When *A_base_* for a particular task was 1.0, accuracy was 0.6 at the rate corresponding to *F_max_*.

For the fitting analysis, we included data with accuracy of >0.6 at any one of the stimulus rates in each task since this fitting function assumed a reasonable level of accuracy at low task rates. According to this accuracy criterion, we excluded two healthy participants (both senior participants) and two PD participants in the movement task, and seven healthy participants (four were senior) and four PD participants in the imagery task. No participants were excluded in the calculation task. We excluded data from a young participant as this participant made almost no mistakes in the movement and calculation tasks, precluding reliable fitting (*F_max_* was estimated to be 6 Hz in the movement task and 5.3 Hz in the calculation task). Fitting did not converge in a movement task in another young participant due to highly variable accuracy. In the end, we obtained *A_base_* and *F_max_* parameters from 42 healthy (22 non-seniors and 20 seniors) and 17 PD participants for the movement task, 39 healthy (21 non-seniors and 18 seniors) and 15 PD participants for the imagery task, and 45 healthy (23 non-seniors and 22 seniors) and 19 PD participants for the calculation task (Extended Data Fig. 2-1). After these procedures, we reconfirmed that basic profiles were comparable between the participants with PD and the controls. After the exclusion, the participants with PD and controls were still matched in age in both movement task (*p* = 0.34; mean age of 11 control males and nine control females, 61.5 ± 8.1 years; mean age of 10 PD males and seven PD females, 64.2 ± 9.3 years) and imagery task (*p* = 0.37; mean age of nine control males and nine control females, 60.6 ± 7.7 years; mean age of eight PD males and seven PD females, 63.3 ± 9.3 years).

*A_base_* and *F_max_* were analyzed with Kruskal–Wallis test and ANOVA, respectively, according to their data distributions. For the pair-wise comparison, we used Mann–Whitney *U* test for the analysis of *A_base_* with non-Gaussian distribution and *t* test for the analysis of *F_max_*with Gaussian distribution. Intertask correlations for *F_max_* were tested between the movement-imagery task pairs (*n* = 38). We ran this analysis to test if the agility parameter (*F_max_*) could capture the correlation of agility between the movement and imagery tasks. Since the time required for performance is correlated between motor execution and motor imagery (Decety and Jeannerod, 1995; Sirigu et al., 1995), the correlation of *F_max_* between the movement and imagery tasks, if found, should support the usefulness of *F_max_* as a measure reflecting processing speed. Furthermore, as the age substantially varied across the healthy participants, we also tested how aging influenced *A_base_* and *F_max_*, using a correlation analysis.

### fMRI experiment: data acquisition

**Figure 3.**
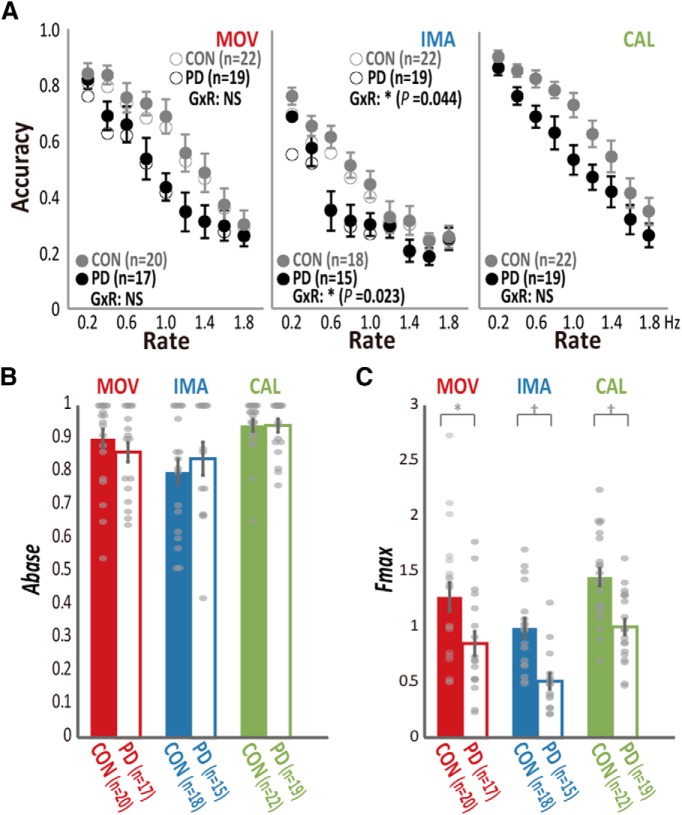
Comparison of the raw accuracy and fitted parameters (*A_base_* and *F_max_*) between participants with PD and controls (CON) in the behavioral experiment. ***A***, Accuracy data averaged across participants in PD (black) and CON (gray) for each rate of the movement (MOV), imagery (IMA), and calculation (CAL) tasks (see Extended Data Fig. 3-1 for the statistical values). Open and filled circles represent the group mean before and after the exclusion of data, respectively, according to the accuracy criterion used for the fitting analysis in the movement and imagery tasks. Although the effects of both GROUP (G) and RATE (R) were significant for all the tasks, only IMA revealed significant group-by-rate interactions (GxR) regardless of the data exclusion, supporting slowing of imagery in participants with PD. Error bars are omitted for the data before the exclusion (open circles) for the clarity of visualization. ***B***, The fitting analysis indicated that the *A_base_* was preserved in participants with PD for MOV, IMA, and CAL tasks. Gray dots represent data from each participant. ***C***, In the fitting analysis, participants with PD showed reduced agility (*F_max_*) in all tasks; *p < 0.05, †*p* < 0.01. Error bars: SEM.

To find the substrates of speeded motor and cognitive performance, rate-correlated brain activity was studied during the three tasks in healthy and PD participants. The tasks were essentially the same as those in the behavioral experiment but were modified to accommodate a block-design fMRI experiment. The participants lay on the scanner bed and viewed visual stimuli rear-projected on a screen through a mirror and wore an MRI-compatible response unit (five buttons) placed beneath the right hand. Throughout the fMRI experiment, electromyography (EMG) was monitored from four hand/forearm muscles with MRI-compatible equipment. An fMRI run (8 min) included eight 30-s blocks of the same task (movement, imagery, or calculation) presented at four rates (0.25, 0.5, 0.75, and 1 Hz) alternated with eight 30-s blocks for fixation baselines. A response prompt was presented for 2.5 s (a response period) at the end of each task block. The participants were encouraged to signal their response to each block with a single button push within the response period, but the response was recorded even after the response period. As the blocks always had the same length, the number of stimuli differed depending on the rate: seven or eight (different across blocks) for 0.25 Hz, 15 for 0.5 Hz, 22 or 23 for 0.75 Hz, and 30 for 1 Hz. We chose these rates since the difference in task performance was most pronounced at the rate of 1 Hz between the PD participants and the healthy controls (Fig. 3*A*). Hence, we used 1 Hz as the highest rate and designed other three rates to approximately cover the rate range used in the behavioral experiment. In an fMRI run, the rate was accelerated and then decelerated for the task blocks. We made the task rate predictable to accommodate for differences in behavioral flexibility among the participants (especially between healthy and PD participants). Two fMRI runs were assigned to each task. The task order followed a Latin square design, but we always started and ended a session using fMRI runs with the movement task to efficiently confirm the quality of EMG. The order of the imagery and calculation tasks differed across participants.

10.1523/ENEURO.0200-17.2017.f3-1 Extended Data Figure 3-1 Statistics from RM-ANOVA for the comparison between participants with PD (*n* = 19) and senior controls (*n* = 22) in the behavioral experiment. Significant results are shown in italic fonts. Download Figure 3-1, TIF file.

We used a 3-T magnet with a standard head coil (GE Medical Systems). Gradient-echo echo planar imaging sensitive to the blood-oxygenation level-dependent signal was used for fMRI with the following parameters: repetition time (TR) = 3 s, echo time (TE) = 30 ms, flip angle = 90°, 64 × 64 matrix, 3.75 × 3.75 × 5-mm voxel-size, and 22 slices covering the whole brain. A three-dimensional structural MRI scan of the brain was acquired using a T1-weighted inversion recovery fast spoiled gradient recall sequence (TR = 8.2 ms, TE = 3.3 ms, inversion time = 725 ms, flip angle = 6°, 256 × 256 matrix, in-plane resolution of 0.97 × 0.97 mm, 124 contiguous axial slices with a thickness of 1.3 mm).

For EMG monitoring, pairs of gold electrodes (Grass Technologies) with an interelectrode distance of ∼3 cm were placed over the right abductor pollicis brevis, abductor digiti minimi, extensor digitorum communis, and flexor digitorum superficialis. EMG data were amplified, digitized (sampling rate = 250 Hz), and saved on a computer. When a lack of EMG activity during the movement task or presence of EMG activity during the imagery or calculation tasks was noticed, the experiment was stopped, and the participants were reminded of the task instructions.

### fMRI experiment: behavioral and EMG data analysis

The accuracy of the responses during the fMRI experiment was analyzed with two-way RM-ANOVA with the task and the rate as within-subject variables in the healthy participants. We used three-way RM-ANOVA with the task and the rate as within-subject variables, and with the GROUP as a between-subject variable, for the comparison between the participants with PD and the controls. We were unable to compute *A_base_* and *F_max_* from the fMRI experiment because of the limited range of task rates and small number of response samples.

EMG signals were first corrected for scanning artifacts using a template subtraction method (Analyzer 2; Brain Products GmbH), followed by bandpass filtering (30-100 Hz), rectification, and normalization to the maximum amplitude in each muscle. The data were averaged across the four muscles (grand-mean normalized EMG) as a time-series for each fMRI run in each participant. We then calculated an integrated EMG (iEMG) parameter that served as a summary of muscle activity for each rate and each task. We tested for the effects of the task and rate on iEMG with RM-ANOVA. Artifact-corrected EMG data for detailed quantitative analyses were available from only 30 (16 seniors) of 38 healthy participants due to technical problems. iEMG was available for quantitative analysis from all participants with PD. The participants with PD and controls were matched in age for the group comparison of the iEMG data (*p* = 0.45; mean age of nine control males and seven control females, 61.9 ± 8.0 years; mean age of nine PD males and six PD females, 64.1 ± 9.8 years).

We analyzed possible changes in tremor during the fMRI experiment in the participants with PD, using a power spectral analysis of the EMG data (3-s windows corresponding to each fMRI volume). After preprocessing, we applied a Fourier transform to produce a power spectrum in the frequency domain. The dominant peak was identified in the tremor frequency (4-8 Hz), and the peak power was measured, providing behavioral data and regressors for fMRI analysis.

### fMRI experiment: preprocessing and first-level statistical analysis

Image preprocessing and statistical analyses were performed with SPM8 (http://www.fil.ion.ucl.ac.uk/spm) in MATLAB (Mathworks, Inc.). After the first four volumes were removed, the time-series fMRI data were aligned in both time and space, spatially normalized to fit to the Montreal Neurologic Institute template, and then smoothed with a Gaussian kernel of 8-mm full-width at half-maximum. A first-line, first-level general linear model analysis for each task tested correlations between fMRI signal changes and a block regressor plus a parametric regressor (modeling 4 task rates) convolved with a canonical hemodynamic response function. The block regressor captured task-related brain activity relative to the baseline, and the parametric regressor captured brain activity correlated with the task rate. A preliminary analysis failed to show differences between the acceleration and deceleration phases of the task rate. Hence, we report the combined results of these two phases. The second-line, first-level analysis modeled task blocks separately for each rate, providing the estimated activity for each rate in each task, which yielded β values for each rate. To reduce the effects of the button-press events and head motion, the corresponding regressors were included in the design matrices. To remove the potential effects of tremor in the PD group, two EMG-derived regressors, the spectral power of the tremor frequency (4-8 Hz) and its log transformation, were also modeled (Helmich et al., 2010). Finally, the data were high-pass filtered (cutoff 512 s) to remove low frequency confounds, and autocorrelation of the data were accommodated with an autoregression model.

### fMRI experiment: second-level statistical analysis

The contrast-weighted β value images were fed into a second stage analysis, with participants treated as a random variable. First, we tested the regional effects of the rate in each task and differences in the rate effect between the tasks in the healthy participants with two-tailed one-sample *t* test. All healthy participants were included to increase the statistical power (*n* = 38). Then, the rate-correlated brain activity was compared between the 15 participants with PD and 18 age-matched healthy participants, using two-tailed two-sample *t* test. For this group-comparison analysis, task-related brain activity was also assessed. Brain activities were reported when they exceeded a height-level significance threshold *p* < 0.05 family-wise error (FWE) corrected for multiple comparisons for the whole-brain search, unless otherwise mentioned. When we had an a priori hypothesis for the region, small volume correction analyses were used based on probability maps of the cortical areas (SPM Anatomy Toolbox), including the primary motor cortex (M1), premotor areas, and inferior frontal cortex (IFC; including Brodmann areas 44 and 45) or the subcortical nuclei (defined by probabilistic diffusion tractography as explained below). In particular, we hypothesized that the M1 and premotor areas would participate in the movement and imagery tasks (Hanakawa et al., 2003) and that the IFC and premotor areas would be involved in the calculation task (Hanakawa et al., 2002). For the nomenclature, we divided the premotor areas into the five following subdivisions: the ventral premotor area, supplementary motor area (SMA), pre-SMA, dorsal premotor area (PMd), and pre-PMd.

### Diffusion MRI-based subcortical gray matter classification

No clear anatomic landmarks are available for discriminating striatal and thalamic subdivisions constituting BTC circuits. The segmentation of striatal and thalamic subdivisions can be objectively achieved with MRI tractography (Behrens et al., 2003; Tziortzi et al., 2013). Hence, we applied MRI tractography to create a map of the striatum and thalamus according to connections with the frontal lobe regions. Diffusion-weighted MR images (DWIs) were obtained on a 3-T scanner with an eight-channel phased-array receiver coil (Siemens Trio). We acquired DWI with twice-refocused, single-shot, spin-echo echo planar imaging (TR = 7900 ms, TE = 80 ms, FA = 90°, slice thickness = 2 mm, matrix size = 96 × 68, FOV = 192 × 192 mm, 68 axial slices). A single acquisition included 81 DWIs (b-value = 1000 s/mm^2^ with different motion-probing gradient directions) and nine non-DWIs (b-value = 0 s/mm^2^). High-resolution T1-weighted and field-map images were also obtained. We used FSL4.1 for diffusion-based subcortical gray matter classification of the striatum (Tziortzi et al., 2013) and thalamus (Behrens et al., 2003). We created nine complementary cortical masks, including the M1 and primary somatosensory areas (areas 1, 2, and 3), premotor areas, and IFC, according to their probabilistic representations in the standard stereotaxic space (Eickhoff et al., 2005). Other cortical masks included the medial prefrontal cortex, orbitofrontal cortex, ventral prefrontal cortex, dorsal prefrontal cortex, fronto-polar cortex, and the rest of the cortex including the occipito-parieto-temporal areas. We estimated tractography paths running between the seed (whole striatum or whole thalamus) and the cortical targets only in the left hemisphere (paths running through the corpus callosum were excluded). The results are expressed as the number of connectivity path samples (5000) at each voxel. We then determined and labeled the cortical subdivision with the highest connectivity to each voxel in the whole striatum and thalamus after scaling connectivity in each cortical region relative to the total. The tractography analysis defined specific BTC circuits in accordance with previous studies (Lehericy et al., 2004b; Verstynen et al., 2012). We used striatal and thalamic subdivisions connected with the IFC, premotor area, and M1 as volumes of interests (VOIs).

## Results

### Behavioral experiment results in healthy participants

In the behavioral experiment, accuracy, which was averaged across the healthy participants, monotonically declined as a function of the task rate in all the tasks, reflecting rate-accuracy trade-off relationship (Fig. 2*A*).

In the fitting analysis at the level of each individual, a sigmoid function fitted to the rate-accuracy trade-off relationship (Fig. 2*B*), yielding parameter estimates reflecting *A_base_* and *F_max_*. Across all healthy participants, *F_max_* was 1.60 ± 0.68 (SD) Hz for the movement task (*n* = 42), 1.33 ± 0.60 Hz for the imagery task (*n* = 39), and 1.48 ± 0.42 Hz for the calculation task (*n* = 45; Fig. 2*C*). The agility parameter did not differ across the tasks (*F*_(2,125)_ = 2.4, *p* = 0.10, ANOVA; *p* = 0.2, Kolmogorov–Smirnov test). To support the hypothesis that *F_max_* is an adequate measure of processing speed, we tested correlation of *F_max_* across the movement and imagery tasks in the 38 healthy participants from whom *F_max_* was available in both tasks. Between the movement and imagery tasks, the agility parameter was strongly correlated (*r* = 0.68, *p* = 3.0 × 10^−6^, *n* = 38), which agreed with a rule dictating the relationship between motor speed and imagery speed (Decety and Jeannerod, 1995; Sirigu et al., 1995). The mean *A_base_* was 0.93 ± 0.11 (SD) for the movement task (*n* = 42), 0.84 ± 0.18 for the imagery task (*n* = 39), and 0.95 ± 0.08 for the calculation task (*n* = 45; Fig. 2*C*). The *A_base_* differed across the tasks (*p* = 0.027, Kruskal–Wallis test was used due to a non-Gaussian distribution, *p* = 2.9 × 10^−9^, Kolmogorov–Smirnov test). The across-task difference in the *A_base_* parameter was due to lower accuracy in the imagery task than in the movement task (*p* = 0.019), which is consistent with a previous report (Hanakawa et al., 2003).

As the age substantially varied across the healthy individuals, we tested how aging influenced *A_base_* and *F_max_*. We found that age was inversely correlated with *F_max_* in both movement (*n* = 42, *r* = −0.58, *p* = 0.0007) and imagery (*n* = 39, *r* = −0.59, *p* = 0.0008) tasks, but not in the calculation task (*n* = 45, *r* = −0.17, *p* = 0.26). These results suggest that aging reduces the agility of motor-related tasks in both physical and cognitive forms, but not agility of mental calculations. Aging did not influence *A_base_* in the movement (*ρ* = −0.30, *p* = 0.06), imagery (*ρ* = −0.22, *p* = 0.24), or calculation (*ρ* = −0.07, *p* = 0.66) task.

### Behavioral experiment results in participants with PD

When we compared group-averaged accuracy data (19 PD and 22 controls) using an RM-ANOVA analysis, we found significant rate-by-GROUP interactions (*F*_(5.0,33)_ = 2.5, *p* = 0.029; Extended Data Fig. 3-1). This interaction can be ascribed to a steeper decline in accuracy as the task became more difficult in the higher rates. Note that at the fastest rates the performance of the two groups was comparable due to excessive task difficulty, so this interaction primarily reflects mid-range task difficulty (Fig. 3*A*). When the accuracy data were analyzed separately for each task with RM-ANOVA, the rate-by-GROUP interactions reached significance in the imagery task (*F*_(4.7,33)_ = 2.38, *p* = 0.04), but not in the movement (*F*_(5.4,33)_ = 1.42, *p* = 0.21) or the calculation task (*F*_(4.7,33)_ = 0.67, *p* = 0.64). When we used RM-ANOVA to reanalyze the data after the application of the exclusion criterion for the fitting analysis, the results were essentially the same (*F*_(4.6,25)_ = 2.78, *p* = 0.023 in the imagery task for 15 PD and 18 controls; *F*_(5.4,33)_ = 2.02, *p* = 0.072 in the movement task for 17 PD and 20 controls; Fig. 3*A*). The lack of statistically significant rate-by-GROUP interactions in the movement and calculation tasks could be explained by interindividual differences in the rate at which individuals started to make mistakes due to insufficient processing time.

When we analyzed the parameters from the fitting analysis, we found clearer evidence for motor and cognitive slowing in PD than the analyses of the group-averaged accuracy data with RM-ANOVA. The PD group compared with the age-matched controls showed reduced agility (*F_max_*) in the movement (*T*_(35)_ = 2.39, *p* = 0.022; 17 PD and 20 controls), imagery (*T*_(31)_ = 4.1, *p* = 0.0003; 15 PD and 18 controls), and calculation tasks (*T*_(39)_ = 3.93, *p* = 0.0003; 19 PD and 22 controls; Fig. 3*B*). Since agility was influenced by age in the analysis of healthy participants, we also ran a supplementary general linear model analysis including age as a covariate. The results supported the reduction of *F_max_* in the PD participants compared with the control participants for the movement task (*F*_(1,34)_ = 4.6, *p* = 0.040), imagery task (*F*_(1,30)_ = 12.7, *p* = 0.001), and also calculation task (*F*_(1,38)_ = 13.3, *p* = 0.001). Conversely, the *A_base_* did not differ between the two groups for the movement (*p* = 0.13, Mann–Whitney *U* test), imagery (*p* = 0.26), and calculation (*p* = 0.28) tasks. Thus, the finding from the fitting analysis indicated that compared with the controls, the present participants with PD had both motor slowing (bradykinesia) and cognitive slowing (bradyphrenia) without impairment of baseline task performance.

### fMRI results in healthy participants

**Figure 4.**
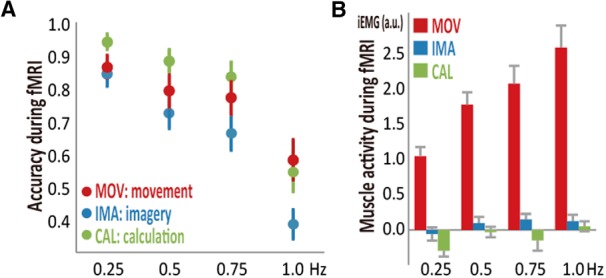
Behavioral and EMG findings from healthy participants during fMRI. ***A***, Accuracy during fMRI (*n* = 38) decreased as a function of the task rate similarly across the movement (MOV), imagery (IMA), and calculation (CAL) tasks (i.e., no task-rate interactions). The imagery task was overall less accurate than the calculation task, while no differences were found between the other task pairs. ***B***, iEMG during fMRI indicated that task rate modulated muscle activity only during the movement task (*n* = 30). Red color indicates MOV, blue indicate IMA, and green indicates CAL.

We analyzed group-averaged accuracy data during fMRI in healthy participants (*n* = 38). Consistent with the behavioral experiment (i.e., no differences in *F_max_* across the tasks), accuracy monotonically decreased as a function of the rate (rate main effect, *F*_(2.4,264.1)_ = 63.1, *p* = 1.0 × 10^−26^, RM-ANOVA) similarly across the three tasks (no task-by-rate interactions, *F*_(4.9,264.1)_ = 1.30, *p* = 0.27; Fig. 4*A*). Also, consistent with the behavioral experiment (i.e., lower *A_base_* in the imagery task), accuracy during fMRI differed across the tasks (task effect, *F*_(2,108)_ = 4.6, *p* = 0.012), with the imagery task showing lower accuracy than the calculation task (*p* = 0.014, Scheffe's posterior test). These results supported an assumption that behaviors were essentially the same between the fMRI and behavioral experiments. During fMRI, muscle activity was modulated by both task and rate (*n* = 30, task main effect, *F*_(1.2,36.2)_ = 91.7, *p* = 1.0 × 10^−20^; rate main effect, *F*_(2.4,69.2)_ = 21.7, *p* = 1.5 × 10^−11^), but the degree of modulation differed across the tasks (task-by-rate interaction, *F*_(3.2,93.3)_ = 10.9, *p* = 4.1 × 10^−9^; Fig. 4*B*). Separate RM-ANOVA indicated that muscle activity correlated with the rate during the movement task (rate main effect, *F*_(1.9,54.0)_ = 21.63, *p* = 2.3 × 10^−7^) but not during the imagery task (*F*_(2.6,76.7)_ = 1.31, *p* = 0.28) or the calculation task (*F*_(2.0,59.3)_ = 3.0, *p* = 0.054), indicating the compliance of the participants with the task instructions.

**Figure 5.**
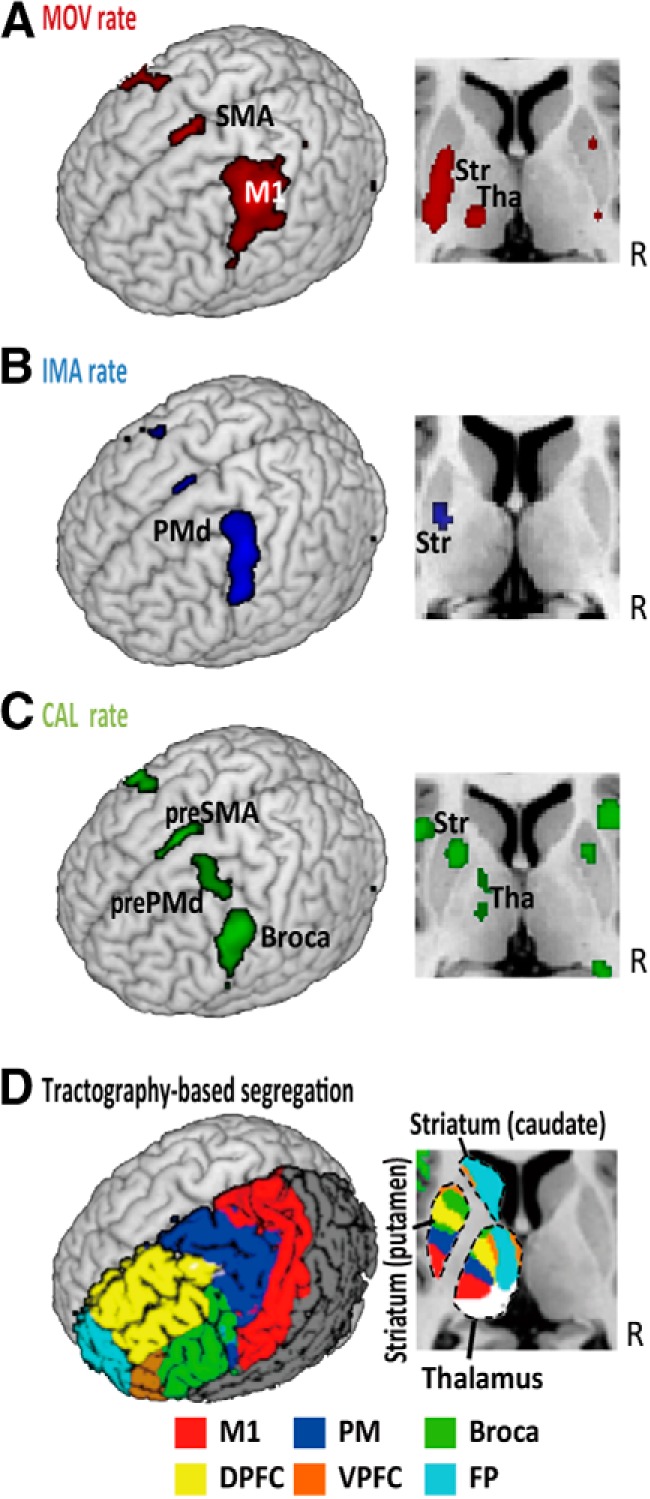
Rate-correlated activity in the BTC circuits. *A*, In healthy participants (*n* = 38), movement (MOV) rate-correlated fMRI activity was found in the primary motor cortex (M1), SMA, posterior striatum (Str), and posterior thalamus (Tha). ***B***, Imagery (IMA) rate-correlated activity was found in the PMd, SMA, and middle Str. ***C***, Calculation (CAL) rate-correlated activity was found in the pre-PMd, pre-SMA, IFC/Broca’s area, anterior Str, and anterior Tha. ***D***, In an independent group of healthy participants (*n* = 15), probabilistic diffusion tractography identified the striatal and thalamic subdivisions connected to the M1, premotor areas (PM), IFC (Broca), ventral prefrontal cortex (VPFC), dorsal prefrontal cortex (DPFC), and fronto-polar cortex (FP).

Rate-correlated fMRI activity was evident in the BTC circuits in a topographical manner depending on the tasks. The movement rate-correlated activity (*p* < 0.05, FWE-corrected) included the M1, caudal premotor areas (PMd and SMA), posterior putamen, and posterolateral thalamus (Table 2, movement rate-correlated activity; Fig. 5*A*). The imagery rate-correlated activity (*p* < 0.05, FWE-corrected) was observed in the left PMd and middle putamen (Table 2, imagery rate-correlated activity; Fig. 5*B*). The calculation rate-correlated activity (*p* < 0.05, FWE-corrected) was observed in the IFC, anterior premotor areas (pre-PMd and pre-SMA), anterior putamen, caudate nucleus, and anterior thalamus (Table 2, calculation rate-correlated activity; Fig. 5*C*). The location of the rate-correlated activity matched the pattern of the cortical connections of the striatum and thalamus identified by the tractography-defined subcortical gray matter classification (Fig. 5*D*). This analysis supported that the movement rate-correlated activity corresponded to the motor striatum and thalamus, the imagery rate-correlated activity to the premotor striatum, and the calculation rate-correlated activity to the IFC-connected (i.e., language-related) striatum and thalamus. Since the behavioral measure of agility showed correlations with aging, we tested the effects of aging onto the rate-correlated fMRI activity in the three tasks. However, we failed to find significant correlations between age and rate-correlated fMRI activity in any of the tasks.

**Table 2.** Activity correlated with task rate in healthy participants (*n* = 38)

	Coordinates				
Activity clusters (functional area)	*x*	*y*	*z*	*T* value	**P*_*FWEcorr*_*
Movement rate-correlated activity					
*Left PMd*	−40	20	60	13.4	6.6 × 10^−16^
Right inferior occipital gyrus (V3)	32	−94	−6	9.31	1.5 × 10^−11^
SMA	0	0	64	9.29	1.6 × 10^−11^
Left inferior occipital gyrus	−44	−74	−12	8.73	8.1 × 10^−11^
Right cerebellar lobule VI	18	−56	−24	8.20	3.8 × 10^−10^
Left cerebellar lobule VI	−40	−62	−24	7.74	1.5 × 10^−9^
Right PMd	50	−4	56	7.44	3.6 × 10^−9^
*Left posterior putamen*	−28	−4	−2	7.12	9.7 × 10^−9^
Left superior parietal 7A	−26	−58	52	7.04	1.2 × 10^−8^
Right posterior putamen	30	0	−6	6.76	0.001
*Left posterior thalamus*	−14	−16	4	6.17	0.003
Imagery rate-correlated activity					
*Left PMd*	−44	−10	62	8.42	2.0 × 10^−10^
Right PMd	54	2	40	8.05	6.0 × 10^−10^
SMA	8	−4	64	7.97	7.6 × 10^−10^
Left inferior occipital gyrus	−34	−94	−6	7.07	1.1 × 10^−8^
Right inferior occipital gyrus (V3)	30	−94	−8	7.12	9.9 × 10^−9^
Left cerebellar lobule VI	−42	−66	−20	6.25	0.003
*Left middle putamen*	−24	−4	12	6.13	0.004
*Left thalamus*	−18	−12	2	5.06	0.025
Left superior parietal 7A	−22	−66	50	5.53	0.020
Right superior parietal 7A	30	−64	52	5.12	0.025
Calculation rate-correlated activity					
Pre-SMA	−4	4	64	11.17	1.0 × 10^−13^
*Left IFC (area 44)*	−48	4	30	10.13	1.6 × 10^−12^
Right inferior occipital gyrus	32	−96	−6	9.89	3.1 × 10^−12^
Left middle occipital gyrus	−30	−96	−8	8.47	1.7 × 10^−10^
Right middle frontal gyrus	54	0	52	8.26	3.2 × 10^−10^
Left cerebellar VIIa	−32	−64	−32	9.45	1.0 × 10^−10^
Right cerebellar VI	34	−62	−26	7.23	7.0 × 10^−8^
Right area 44	44	4	24	6.91	1.9 × 10^−8^
Right angular gyrus	32	−70	36	6.82	0.001
Left angular gyrus	−42	−42	36	6.76	0.001
*Left putamen*	−22	−4	16	6.74	0.001
*Left caudate nucleus*	−16	−4	20	6.32	0.002
Left pre-PMd	−30	−4	56	6.40	0.002
*Left anterior thalamus*	−14	−12	8	6.38	0.002
Right anterior putamen	24	10	6	6.08	0.004

Nodes of the BTC circuits in the left hemisphere are shown in italic fonts. *P_FWEcorr_*: *p* value corrected for multiple comparisons (FWE) in terms of a height threshold.

**Figure 6.**
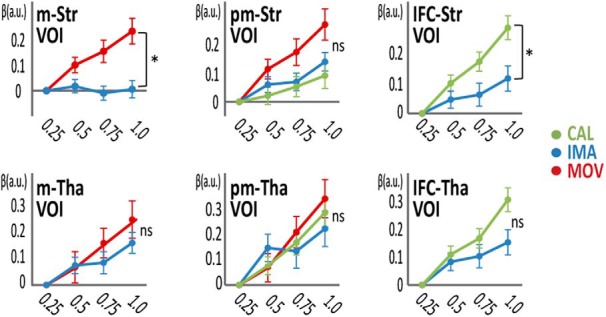
Tractography-based VOI analysis of rate-correlated activity in the striatum (Str) and thalamus (*n* = 38). Tractography-defined motor Str (m-Str) showed greater rate-correlated activity for the movement (MOV) task than for the imagery (IMA) task in the β-value plot (relative to the 0.25-Hz activity) against task rate. The area of the Str connected with the IFC (IFC-Str) showed greater rate-correlated activity for the calculation (CAL) task than for the imagery task. The premotor-connected Str (pm-Str) did not show task specificity of the rate-correlated activity. None of the thalamic VOIs (m-Tha, pm-Tha, or IFC-Tha) showed task specificity of the rate-correlated activity. Red color indicates MOV, blue indicate IMA and green indicates CAL; *p < 0.05. Error bars: SEM; a.u.: arbitrary units.

The rate-correlated activity was compared between the tasks to determine its specificity, with aid from tractography to define the striatal and thalamic VOIs. Comparing the movement to imagery tasks, we found that the M1, SMA, posterior putamen, and cerebellum showed activity that was more strongly correlated with the movement rate than with the imagery rate (Table 3, movement rate-correlated activity > imagery-rate correlated activity). The tractography-defined VOI analysis confirmed the task-specificity of the movement rate-correlated activity in the M1-connected striatum but not in the M1-connected thalamus (Fig. 6). We failed to find activity more strongly correlated with imagery rate than with movement rate in both the whole-brain analysis and VOI analysis. For the comparison between the imagery and calculation tasks, the left PMd revealed activity that was more strongly correlated with imagery rate than with calculation rate (Table 3, imagery rate-correlated activity > calculation-rate correlated activity). Conversely, the IFC, pre-SMA and caudate nucleus showed activity that was more strongly correlated with calculation rate than with imagery rate (Table 3, calculation rate-correlated activity > imagery-rate correlated activity). The VOI analysis confirmed the calculation task-specificity of the rate-correlated activity in the IFC-connected striatal VOI but not in the IFC-connected thalamic VOI (Fig. 6). The striatal VOIs and thalamic VOIs connected with premotor areas showed rate-correlated activity similar across all three tasks. This finding was replicated even when we retrieved all the three types of rate-correlated activity in the peak single voxel of the imagery rate correlated activity in the striatum (data not shown).

**Table 3.** Comparison of rate-correlated activities across tasks in healthy participants (*n* = 38)

	Coordinates				
Activity clusters (functional area)	*x*	*y*	*z*	*T* value	**P*_*FWEcorr*_*
Movement rate-correlated activity > imagery-rate correlated activity					
Right cerebellar lobule VI	28	−50	−28	6.17	0.004
*Left M1*	−42	−24	52	5.61	0.017
*Left posterior putamen*	−32	−16	2	4.11	0.002_svc_^‡^
SMA	2	−14	60	3.81	0.096_svc_^†^
Imagery rate-correlated activity > calculation-rate correlated activity					
*Left PMd*	−38	−20	60	4.40	0.023_svc_^†^
Calculation rate-correlated activity > imagery-rate correlated activity					
*Left IFC (area 44)*	−46	6	20	5.69	0.014
*Left caudate head*	−18	4	18	3.48	0.048_svc_^‡^
Pre-SMA	−6	8	64	3.92	0.075_svc_^†^

Nodes of the BTC circuits in the left hemisphere are shown in italic fonts. *P_FWEcorr_*: *p* value corrected for multiple comparisons (FWE) in terms of a height threshold. svc^‡^: significant for small volume correction within connectivity-based striatal masks; svc^†^: significant for small volume correction within anatomically defined premotor areas based on a probability map.

Overall, these analyses supported task-specific rate-correlated activity in the M1- and IFC-connected striatum to respond to speeded movement and speeded calculation, respectively. However, despite the PMd showing more rate-correlated activity in the imagery task than the calculation task, the premotor-connected putaminal VOI showed rate-correlated activity not only for the movement and imagery tasks, but also for the calculation task.

### fMRI experiment: comparisons between PD and elderly controls

We compared behaviors and brain activity during the fMRI experiment between the 15 participants with PD and 18 matched controls to find the neural correlates responsible for motor and cognitive slowing identified in the fitting analysis of the behavioral experiment. However, since not all the PD and control participants reported above participated in the fMRI experiment, we reanalyzed *F_max_* of the participants in the fMRI experiment. The results confirmed that the PD participants had imagery slowing (*T*_(29)_ = 4.0, *p* = 0.004; *F_max_* not available from two controls and two PD participants) and calculation slowing (*T*_(31)_ = 4.9, *p* = 0.0003), and marginally significant motor slowing (*T*_(31)_ = 2.1, *p* = 0.053). Also, these participants with PD showed mild to moderate bradykinesia indexed by the bradykinesia subscale of the unified PD rating scale (UPDRS; Extended Data Table 1-1).

**Figure 7.**
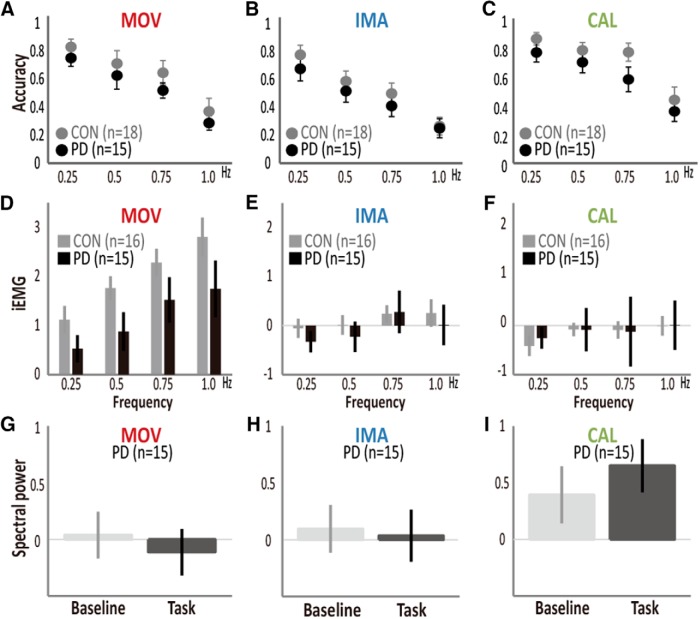
Behavioral and EMG findings in participants with PD (black) and controls (light gray). During the fMRI experiment, accuracy in the movement (MOV; ***A***), imagery (IMA; ***B***), or calculation (CAL; ***C***) task did not differ between the participants with PD (*n* = 15) and controls (*n* = 18). See Extended Data Figure 7-1 for the statistical values. ***D***, Compared with healthy senior participants (*n* = 16; data were not available from two controls due to technical problems), participants with PD (*n* = 15) showed iEMG activity (arbitrary units) responding to the movement rate to a similar degree. iEMG did not show GROUP-by-rate interactions during the imagery (***E***) or calculation task (***F***). Power spectral analysis (4-8 Hz) of EMG data (arbitrary units) in participants with PD (*n* = 15) in the movement (***G***), imagery (***H***), and calculation (***I***) tasks. No differences in the EMG spectral power were found between the task and rest periods, suggesting that tremor did not change in the task condition. Error bars: SEM.

Task performance during fMRI (Fig. 7*A–C*) showed a trend toward differences between the two groups for the three tasks overall (main effects of GROUP; *F*_(1,31)_ = 3.1, *p* = 0.087). Yet, rate-by-GROUP (*F*_(2.2,29)_ = 0.31, *p* = 0.755) or task-by-rate-by-GROUP interactions (*F*_(4.5,26)_ = 0.19, *p* = 0.92) were not significant (Extended Data Fig. 7-1 for the statistical values). Hence, slowing of performance was not detected in PD directly through the responses during the fMRI experiment. Muscle activity was compared across the groups (16 senior and 15 PD participants) in each task because of the task-by-GROUP interactions (*F*_(1.6,28)_ = 5.1, *p* = 0.015, RM-ANOVA; Fig. 7*D-F*). Participants with PD showed lower muscle activity during movement, but the movement rate similarly influenced iEMG between the groups (no GROUP-by-rate interactions, *F*_(1.7,27)_ = 0.35, *p* = 0.67). iEMG during imagery (*F*_(2.8,27)_ = 0.54, *p* = 0.65) or calculation task (*F*_(2.1,27)_ = 0.13, *p* = 0.89) did not show the GROUP-by-rate interactions. EMG spectral analysis in PD (*n* = 15) failed to show tremor-related EMG power changes between the task and baseline periods for the movement (*T*_(14)_ = 2.09; *p* = 0.06), imagery (*T*_(14)_ = 0.66; *p* = 0.52) and calculation tasks (*T*_(14)_ = 1.16; *p* = 0.26; Fig. 7*G-I*). Overall, decline in processing speed during fMRI was latent in the participants with PD. Notably, the differences in task performance and muscle activity (including tremor) alone did not account for the differences in rate-correlated activity between the groups as will be described next.

10.1523/ENEURO.0200-17.2017.f7-1 Extended Data Figure 7-1 Statistics from RM-ANOVA for the comparison between participants with PD (*n* = 15) and senior controls (*n* = 18) in the fMRI experiment. Significant results are shown in italic fonts. Download Figure 7-1, TIF file.

**Figure 8.**
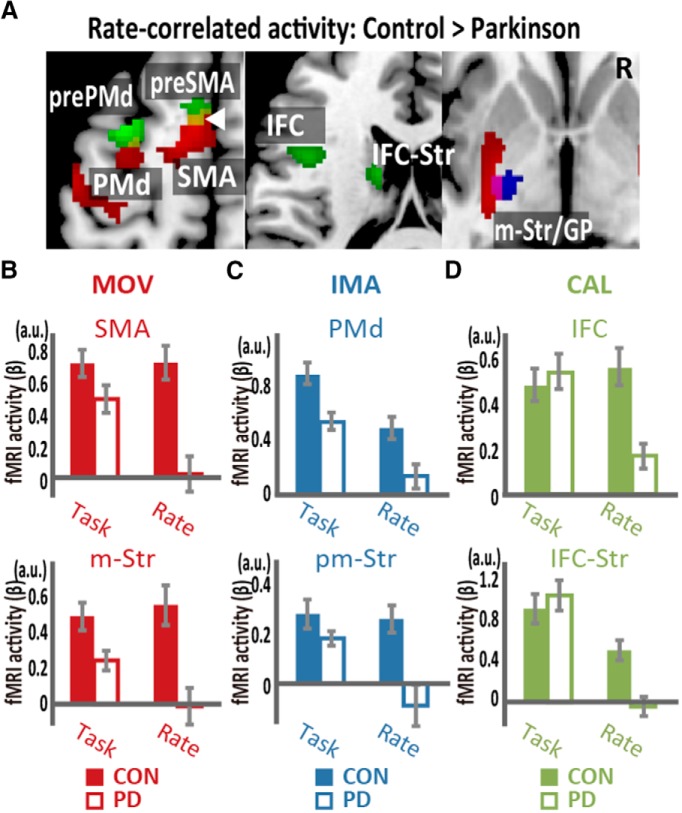
Comparison of rate-correlated activity between participants with PD and controls (CON). ***A***, Participants with PD showed reduced rate-correlated activity in the SMA, PMd, and motor striatum/globus pallidus (m-Str/GP) for the movement task (MOV, red), in the mStr/GP for the imagery task (IMA, blue), and in the IFC, pre-SMA, pre-PMd, and a part of the IFC-connected caudate head (IFC-Str) for the calculation task (CAL, green). The yellow region (indicated by white arrowhead) represents the limited overlaps of the reduced activity between the calculation rate-correlated activity and movement rate-correlated activity. ***B***, The task-related and rate-correlated fMRI activities (β) in the SMA and m-Str are shown for the two groups during the movement task. ***C***, Task-related and rate-correlated fMRI activities in the PMd and pm-Str during the imagery task. ***D***, Task-related and rate-correlated fMRI activities in the IFC and IFC-Str during the calculation task.

Finally, we compared rate-correlated brain activity between the participants with PD (in the off state) and the age-matched controls. The analysis showed a lower level of rate-correlated activity in the task-specific BTC circuits for each of the three tasks in the PD group than in the control group (Fig. 8; Table 4), whereas task-related activity in the BTC circuits did not differ in any task between the groups (Fig. 8*B–D*). Specifically, the PD group showed reduced movement rate-correlated activity in the M1, caudal premotor areas (PMd and SMA), and motor striatum (Fig. 8*A*,*B*). The reduction of imagery rate-correlated activity was detected in the premotor BTC circuit (Fig. 8*A*,*C*). Furthermore, the PD group showed reduced calculation rate-correlated activity in the IFC and the caudate nucleus, constituting the language BTC circuit plus the anterior premotor areas (pre-PMd and pre-SMA; Fig. 8*A*,*D*). Importantly, little overlap was found between these dysfunctional BTC circuits, indicating that hypofunction of the task-specific BTC circuits underlies cognitive and motor slowing in PD.

**Table 4.** Differences in task-related activity and rate-correlated activity between senior control participants (*n* = 18) and participants with PD (*n* = 15)

PD vs CON	Coordinates					
Activity clusters (functional anatomy)	*x*		*y*	*z*	*T* value	**P*_*FWEcorr*_*
Imagery task-related (CON > PD)						
*Left PMd*	−30		−8	48	5.55	0.042
Movement rate-correlated (CON > PD)						
Left visual areas	−36		−92	4	5.68	0.005
Right visual areas	48	−52		−16	5.68	0.005
*Left SMA*	−10	−2		60	4.75	0.045
Right cerebellum	12	−56		−22	4.75	0.045
*Left PMd*	−46	18		56	4.47	0.039_svc_^‡^
*Left posterior striatum-globus pallidus*	−24	−14		−2	4.10	0.039_svc_^†^
Imagery rate-correlated (CON > PD)						
*Left posterior striatum-globus pallidus*	−28	−10		−4	4.29	0.049_svc_^†^
Calculation rate-correlated (CON > PD)						
Left pre-PMd	−30	2		62	5.91	0.001
Left parietal cortex	−44	−44		36	4.77	0.024
Left pre-SMA	−6	14		60	4.21	0.046_svc_^‡^
*Left caudate head*	−14	−4		20	4.29	0.002_svc_^†^
*Left IFC*	−50	12		30	3.7	0.022_svc_^†^

Nodes of the BTC circuits in the left hemisphere are shown in italic fonts. CON: controls; svc^‡^: significant for small volume correction within rate-correlated areas for each task from the whole healthy participants group; svc^†^: significant for small volume correction within anatomically defined cortical areas or diffusion-based classification of subcortical nuclei.

## Discussion

We developed a series of movement, motor imagery, and calculation tasks that allow us to measure speed of thinking (set by task rate) while eliminating potential motor confounds. With a conventional analysis with RM-ANOVA, we only confirmed slowing of motor imagery in PD. However, by applying nonlinear fitting to the rate-accuracy trade-off function, we found that participants with PD also had slowing in movement and calculation tasks, with preserved *A_base_*. We next explored the neural correlates responding to the rate demands of the motor, imagery and calculation tasks in healthy participants, revealing the involvement of partially overlapping yet segregated BTC circuits in the speeded performance of the motor, imagery and calculation tasks. Finally, we found that reduced rate-correlated activity in the motor, premotor and language BTC circuits was associated with slowing of movement, imagery and calculation tasks in PD, respectively.

### Motor and cognitive agility in healthy participants and PD

Previous studies reported conflicting results regarding cognitive slowing in PD. These studies used a memory scanning task (Wilson et al., 1980; Howard et al., 1994), various reaction time tasks (Pillon et al., 1989; Cooper et al., 1994; Duncombe et al., 1994; Pate and Margolin, 1994; Lee et al., 1998, 2003; Vlagsma et al., 2016), “processing speed” components in cognitive batteries (Helscher and Pinter, 1993; Muslimovic et al., 2005; Jokinen et al., 2013), and a rate-accuracy trade-off paradigm (Sawamoto et al., 2002). Some of these studies used a measure of processing speed defined as the speed at which an individual completes a basic cognitive task, such as item identification or simple discriminations (Deary et al., 2010). However, conventional processing speed measures are likely affected by task difficulty, which could explain why cognitive slowing studies may find decreases in task performance rather than decreases in processing speed in PD (Helscher and Pinter, 1993). We used nonlinear fitting to ensure that the *A_base_* parameter absorbed the differences in difficulty across tasks and that the *F_max_* parameter sensitively detected processing speed.

Importantly, the agility parameter was correlated between the movement task and the imagery task in healthy participants. This observation replicates the finding showing that the amount of time required to complete a task is correlated between motor execution and motor imagery (Decety and Jeannerod, 1995; Sirigu et al., 1995), warranting *F_max_* as a measure of agility across motor and cognitive tasks.

In bradyphrenia studies, another important factor is whether participants with PD are “on” or “off” dopamine medications because dopamine medications improve cognitive slowing, depending in part on the task and baseline dopamine levels (Cools et al., 2003). Here, we studied PD participants in a relatively low dopamine state and found the coexistence of bradykinesia and bradyphrenia, lending support for the role of dopamine in agility across behavioral domains. The present approach demonstrated that compared with the controls, the participants with PD had preserved *A_base_* across the three tasks, indicating that basic cognitive impairment was minimal in the PD group. By contrast, the reduced agility (*F_max_*) indicated both bradykinesia and bradyphrenia. Slowing of imagery agility has been replicated in previous studies (Dominey et al., 1995; Heremans et al., 2011), and slowing of calculation agility is consistent with slowing of verbal mental operation (Sawamoto et al., 2002) in PD. The present method can assess agility across different task domains after removing the effects of task difficulty, thereby successfully providing evidence of the coexistence of bradykinesia and bradyphrenia. Thus, the present approach extended the results of a previous study in which bradyphrenia was assessed, using speed-accuracy trade-off (Sawamoto et al., 2002).

Cognitive impairment in nondemented PD is widely accepted, although slowing/absence of movement (bradykinesia/akinesia) is the classic hallmark. However, it has been difficult to pinpoint the mechanisms of cognitive dysfunctions in PD, which is now recognized as a multi-system degenerative disease that potentially involves cortical Lewy body pathology and multiple cortico-subcortical circuit pathologies (Hanganu et al., 2015). A few different mechanisms may be responsible for different types of cognitive impairment including, but not limited to, executive, visuospatial and language dysfunctions and cognitive slowing in PD.

### Segregation of BTC circuits representing rate-correlated activity during movement, imagery, and calculation tasks

Little is known about the neural substrates underlying the control of cognitive processing speed. In fact, few studies have addressed the effects of task rate on brain activity during a cognitive task. The present rate-accuracy trade-off paradigm successfully elucidated the axes of task-relevant neural networks. Here, the results from healthy participants showed rate-correlated activities that revealed the core neural architecture underlying the tasks. In particular, speeded performance of the three tasks recruited the BTC circuits, which are characterized by both topographical segregation and convergence.

For topographical segregation, the movement rate-correlated activity in the motor BTC circuit extends previous observations regarding the effects of movement rate on brain activity (Sadato et al., 1996; Lutz et al., 2005; Hayashi et al., 2008). The comparison of the movement rate-correlated activity with the imagery rate-correlated activity indicates that the involvement of the M1 and M1-connected striatum is greater in movement execution than in motor imagery. Organized in parallel to this motor BTC circuit, the language BTC circuit showed activity sensitive to the rate of a mental calculation task. Increased activity in the fronto-parietal cortical areas, including the IFC, has been reported in association with calculation demands such as the complexity of calculation (Gruber et al., 2001; Fedorenko et al., 2012). However, to our knowledge, the present study is the first to characterize the involvement of the language BTC circuits in rate demands on mental calculation. Although the IFC represents both language-specific and domain-general regions (Fedorenko et al., 2012), the greater IFC activity for calculation rate than for imagery rate suggests that the present IFC site is not so domain general.

Along with the language BTC circuit, the calculation rate-correlated activity was observed in the pre-SMA and pre-PMd. Pre-PMd activity is often reported during a calculation task and is probably related to an spatial aspect of mental calculation (Hanakawa, 2011). The pre-SMA is included in an extended language network (Dick et al., 2014). In fact, previous research has shown that language-related tasks, such as verbal working memory tasks and mental calculation tasks, coactivate the pre-SMA and IFC (Hanakawa et al., 2002). Anatomically, the pre-SMA and IFC are connected through a fiber tract (“frontal aslant tract”; Catani et al., 2012). Moreover, rostral premotor areas project to adjacent sections of the striatum, bridging over the caudate nucleus and putamen (Tachibana et al., 2004), which may overlap the cortico-striatal projection from the IFC (Fig. 5*D*). Thus, the pre-SMA, IFC, and basal ganglia likely constitute an extended language/calculation-related network. Consistent with this idea, nonfluent aphasia can follow damage to the IFC (Broca’s aphasia), pre-SMA (Hertrich et al., 2016), and basal ganglia (Kuljic-Obradovic, 2003).

Motor imagery is a unique cognitive ability, which shares underlying mechanisms with physical movement. Although rate is one of the factors defining a motor imagery task (Hanakawa, 2016), little is known regarding the neural architecture that responds to the rate of motor imagery. To our knowledge, the present study is the first demonstration of the neural substrates for speeded motor imagery. The comparison of imagery rate-correlated activity with movement or calculation rate-correlated activity advanced knowledge regarding the neural correlates of motor imagery. The areas showing imagery rate-correlated activity were essentially included in those showing movement rate-correlated activity. Particularly, the rate-correlated activity of the movement and imagery tasks overlapped in the premotor BTC circuit, replicating the shared substrates between these tasks (Gerardin et al., 2000; Hanakawa et al., 2003, 2008).

### Convergence of rate-correlated activity across BTC circuits

Although the central axes of the BTC circuits are topographically segregated, emerging evidence indicates substantial overlap and convergence across the BTC circuits, especially in the limbic and cognitive domains (Averbeck et al., 2014). However, it remains unclear what kind of information is integrated in the convergence within the BTC circuits. The present analysis of rate-correlated activity during different tasks uncovered converging properties between imagery rate- and calculation-rate correlated activity in the BTC circuits. The PMd showed greater rate-correlated activity during imagery than during calculation. The PMd is one of the most repeatedly reported sites of activation during motor imagery (Hanakawa, 2016), reinforcing the central role of the PMd in motor imagery. Nevertheless, we unexpectedly found substantial calculation rate-dependent activity in the subcortical parts of the premotor-connected BTC circuit. This finding may reflect the integration or funneling processes within the BTC circuits (Haber, 2003), since mental calculation and motor imagery may share a cognitive process called “amodal” imagery (Hanakawa et al., 2004). Future studies are necessary to test this interpretation, however.

### Dysfunctions of parallel BTC circuits as the underlying pathophysiology of motor and cognitive slowing in PD

PD pathology affects dopamine neurons, which form the mesolimbic and nigro-striatal projections. Through these two circuits, dopamine may play a dual role in regulating effortful cognition (Westbrook and Braver, 2016). In fact, a long-standing topic of debate is whether cognitive disturbance in PD results from dysfunction of the prefrontal-limbic system following mesolimbic damage or from BTC dysfunction following nigro-striatal damage (Owen et al., 1998; Dagher et al., 2001; Mattay et al., 2002; Monchi et al., 2007; Sawamoto et al., 2007; Jokinen et al., 2013). Although both are possible mechanisms, the present study revealed that both bradykinesia and bradyphrenia were associated with dysfunctions of the BTC circuits involving the dorsal striatum and posterior frontal cortices, not the prefrontal-limbic circuitry. In the motor domain, we replicated the results of many studies that attributed the dysfunctional motor BTC circuit to the pathophysiology of bradykinesia (Playford et al., 1992; Herz et al., 2014; Michely et al., 2015). In the cognitive domains, the present study provided novel evidence that cognitive slowing in the imagery and calculation tasks can be ascribed to dysfunctions of the premotor and language BTC circuits, respectively. Altogether, our findings provide a perspective showing that cognitive slowing in distinct behavioral domains is associated with dysfunctions of distinct BTC circuits in a parallel manner. However, this finding does not mean that motor slowing and cognitive slowing should show similar severity at a disease stage. The depletion of nigro-striatal dopamine starts from the motor striatum, demonstrating that motor slowing prevails at least at an early stage of the disease. Dopamine depletion later affects the anterodorsal cognitive striatum (Kish et al., 1988), explaining lagged and milder cognitive slowing compared with motor slowing in typical cases with PD.

The BTC circuits are implicated in a variety of tasks including, but not limited to, behavioral initiation/switch (Crinion et al., 2006), reinforcement/conditional learning (Doya, 2008; Koralek et al., 2012), sequential behaviors (Brotchie et al., 1991; Desrochers et al., 2010), time-constrained decision-making (Glimcher, 2011; Wan et al., 2011), and motor control. Nevertheless, the type of neural computation that is conducted within the BTC circuits remains unclear. Whether this computation is specific to each task-specific BTC circuit or based on a universal principle across different BTC circuits is also unknown. Although answering this fundamental question is beyond the scope of the present study, we have provided evidence that may ultimately contribute to the answer. We showed that the segregated axes of BTC circuits participated in meeting the demands for speeded performance across different tasks and that hypofunction of the cognitive and motor BTC circuits accompanied cognitive and motor slowing following dopamine loss. These findings suggest that task-specific BTC circuits may share a function to boost speeded performance of the respective task in a dopamine-dependent manner.

Given the role of dopaminergic modulation of segregated BTC circuits in speeded performance across tasks, a crucial question is how a single neurotransmitter, dopamine, modulates the functions of distinct circuits relevant to different functions. Indeed, this question has drawn much attention recently (Matsumoto 2015; Westbrook and Braver, 2016). Regarding the relationship between dopamine and behavior, an influential concept is the reward theory (Schultz et al., 1997). In this theory, a phasic release of dopamine codes “reward prediction error” signals by which an organism implicitly knows the values of a given stimulus or a behavior in the form of the likelihood of obtaining rewards. Phasic dopamine release based on reward expectation should influence the motivational level for behavior. Accordingly, abnormal dopamine release in PD could result in impairment of the motivation for behavior across different tasks, especially when the tasks are associated with reward. However, this theory cannot be easily applied to the interpretation of the present paradigm in which no feedback was provided after each trial. Furthermore, the dopaminergic system that conveys such value signals is located in the ventromedial midbrain, which projects to the ventral striatum and ventromedial prefrontal cortex (Matsumoto and Hikosaka, 2009). Conversely, the present study revealed the relevance of the dorsal striatum and posterior frontal cortex to speeded performance across motor and cognitive tasks.

In the dorsal striatum, a tonic increase of dopamine is suggested to invigorate actions to finalize a solution (Westbrook and Braver, 2016). Indeed, the present task design placed an emphasis on quick and robust execution of stimulus-response/operation linkage toward a solution; PD participants showed cognitive and motor disturbance, especially when speeded performance was required. A previous study has also proposed that the dorsolateral dopaminergic neurons carry information regarding the salience of stimuli (Matsumoto and Hikosaka, 2009). Frequent processing of stimuli may activate distinct BTC circuits if the saliency of a stimulus is defined contextually in the form of stimulus-response linkage. Consistently, striatal releases of dopamine have been proposed to regulate the gating policy to determine what kind of signal is transmitted to the frontal cortex (O'Reilly and Frank, 2006). The striatum may create a response bias toward enhanced performance (Lauwereyns et al., 2002). It is thus plausible that the BTC circuits invigorate the functions of task-specialized frontal executive regions, and this function may rely on dopamine release in the dorsal striatum. This function seems particularly relevant to the present tasks, all of which require step-by-step sequential stimulus-response/operation linkage toward a solution. Such driving functions of the BTC circuits likely come into play at a point where the efficiency of the frontal executive regions alone cannot handle a task. We propose that the BTC circuits control the rate demands of distinct cognitive and motor tasks in a homologous manner, indicating a ubiquitous function across the BTC circuits passing through the dorsal striatum.

### Limitations of the study

Although the fitting analysis helped the detection of bradykinesia and bradyphrenia in PD, this analysis was not applicable to the data with low or too high performance regardless of the task rate, or to the data with highly variable accuracy. Low performance was especially problematic in the imagery task probably because an ability of motor imagery substantially differs even across healthy participants (Kasahara et al., 2015). However, the fitting analysis was applicable to all data in the calculation task. It is likely that the present rate paradigm is suitable to an overlearned task like simple arithmetic.

The behavioral experiment and fMRI experiment employed essentially the same tasks and the parametric design, but the number of stimuli for each block was varied to accommodate for block-design fMRI with a single response at the end of each block. This is an inherent limitation in imaging studies employing different task/stimulus rates across blocks. Hence, it should be noted that rate-correlated activity also reflects differences in the number of stimuli and trials across blocks.

We failed to capture bradykinesia or bradyphrenia directly from the behavioral data during the fMRI experiment. This is not surprising since a similar analysis of the behavioral data only detected evidence for slowing in the imagery task (Fig. 3*A*). The comparable performance between the two groups should make the interpretation of the fMRI findings rather simple since the differences in brain activity do not likely result from differences in performance (rather than underlying pathophysiology). However, clinical assessment (UPDRS bradykinesia subscale) and the *F_max_* analysis from the behavioral study indicated that the same participants with PD had both bradykinesia and bradyphrenia compared with the controls. Therefore, we interpreted that the fMRI findings from the PD versus control groups reflect potentially bradykinetic and bradyphrenic symptoms in PD.

## Conclusion

We confirmed the coexistence of motor slowing and cognitive slowing in PD, using a novel approach based on a rate-accuracy trade-off paradigm. The imaging findings indicated a function spanning over the multiple BTC circuits to invigorate both motor and cognitive frontal regions, thereby allowing for enhanced speeded performance regardless of the task domains. Furthermore, hypofunction of specific BTC circuits is associated with cognitive and motor slowing in PD.

## Synthesis

Reviewing Editor: Jörg Schulz, RWTH Aachen University Decisions are customarily a result of the Reviewing Editor and the peer reviewers coming together and discussing their recommendations until a consensus is reached. When revisions are invited, a fact-based synthesis statement explaining their decision and outlining what is needed to prepare a revision will be listed below. The following reviewer(s) agreed to reveal their identity: Li Li, Guy Yona

One reviewer was satisfied with the changes, the other reviewer still has a number of important comments, that will be very helpful for the revision and should be taken into account:

There are no major issues now, but the writing is not quite clear and there are many typos and inconsistent expressions. I tried to ignore them but they just popped out and were quite annoying. If the authors were more carefully, I could have put more energy to provide feedback on the content. I have to say my attention was mainly drawn to the typos and inconsistent expressions when I read the revision. In addition, one figure is missing. See my detailed comments below:

1. Page 14, line 347, where is Figure 2D?

2. Page 8, line 186, it seemed that “in the movement ask” was missing after “we excluded ... participants”.

3. For all the statistical results, it's better to report the degree of freedom

4. Page 9, para 2, the description of fMRI experiment is still not quite clear to me. e.g., line 218, I'm wondering whether “an fMRI run (8 min)” = eight 30-s blocks of same task + eight 30-s blocks of fixation?

5. Page 15, para 2, the explanation of statistical results is not clear to me. Why does the interaction indicate the overall reduced processing speed in PD? The interaction just indicates that the data trend for the imagery task are different from those for the movement and the calculation task. It seemed that in general the performance of PD patients and HC were similar at the low and high frequencies. PD patients were less accurate at the middle frequencies than HC. The converge of performance of PD and HC at high frequencies were earlier for the imagery task than for the other two tasks. In RM-ANOVA analyses separate for each task, whether there are main effect of group for the movement task and the calculation task?

6. Page 16, line 394, “These results supported an assumption that behaviors were essentially the same between the fMRI and behavioral experiments”. However, it seemed that the results of accuracy in healthy participants were not shown.

7. Page 38, line 903, should be “due to”; line 905, please delete “Error bars: S.E.M”

8. Page 38, line 917, please delete “)”

9. Page 18, line 447, here it's better to report the score of specific bradykinesia items in UPDRS-motor, not UPDRS-motor itself. UPDRS-motor>0 could be due to tremor and rigidity etc.

10. In the paper, sometimes the statistical analysis was conducted on what variable is vague. For example, page 15, line 364, it's clearer to say “group-averaged accuracy data”.

11. Figure 2C, please change the order of Abase and Fmax to match the order in text.

12. Figure 2C, “*” indicates the sig. difference between MOV and IMA, thus it should be put between MOV and IMA (like those in Figure 3C) , not on the top of IMA bar

13. Figure 2, 3, 4, & 7, please stick to one type of figure for performance accuracy, i.e., either using Line chart (now in figure 2&3) or Scatter (now in figure 4&7) to plot performance accuracy.

14. Figure 3A, “GxR”, “Gxr” or “G*R”, please stick to one expression

15. Figure 3A, no error bars for the group means before the exclusion of data?

16. Figure 6, please use legend symbols to indicate tasks, like those in Figure 2 & 3

17. Figure 6, “‡” or “*”, please stick to one symbol to indicate p<0.05

18. Figure 7, what is Y-axis for in Figure 7G-7I? In addition, it would be clearer if the authors use legend symbols to indicate groups in the figure and add titles to indicate the tasks (i.e., MOV for figure A,D,G; IMA for figure B,E,H; and CAL for figure C,F,I)

19. Table 4, for the task-related activity, only Imagery task-related was reported, how about the other two tasks? They were not shown because of non-significant results? However, for the rate-related activity, all the nonsignificant results were shown in the same table.

20. Table 6, line 874, please delete “6”
