# Supplementary material for: A Common Function of Basal Ganglia-Cortical Circuits Subserving Speed in Both Motor and Cognitive Domains
Source: eNeuro. 2017 Dec 8;4(6):ENEURO.0200-17.2017. doi: 10.1523/ENEURO.0200-17.2017 (PMC5783269; doi:10.1523/ENEURO.0200-17.2017)
Supplement: Extended Data Table 1-1 — Full profiles of the subgroups of healthy participants and participants with Parkinson's disease (PD) in the fMRI experiment. Download Table 1-1, DOCX file. [file sup_enu-eN-NWR-0200-17-s02.docx]

**Extended Data Table**

**Table 1-1: Full profiles of the subgroups of healthy participants and participants with Parkinson’s disease (PD) in the fMRI experiment**

|  | Healthy adults (n = 38) | | Participants with PD (n = 15) | Statistics (senior control vs. PD) |
| --- | --- | --- | --- | --- |
|  | Non-senior (n = 20) | Senior control for PD (n = 18) |  |  |
| Age (mean±s.d) yrs | 29.6±8.6 | 62.2±8.2 | 64.1±9.8 | *T_(31)_* = 0.59, *P* = 0.56^†^ |
| Male:Female | 11:9 | 10:8 | 9:6 | χ^2^ = 1.06, *P* =0.79^‡^ |
| EHI (mean±s.d.) | 0.9±0.1 | 0.91±0.015 | 0.93±0.20 | *T_(31)_*= 0.36, *P* = 0.72^†^ |
| Education (mean±s.d.) yrs | 15.9±4.0 | 16.5±3.0 | 18.3±2.7 | *T_(31)_* = 1.7, *P* = 0.09^†^ |
| MMSE (mean±s.d.) | 29.7±0.5 | 29.5±0.8 | 29.5±0.7 | *T_(31)_* = 0.12, *P* = 0.90^†^ |
| Experience | N = 13 | N = 10 | N = 9 | χ^2^ = 3.0, *P* = 0.39^‡^ |
| Hoehn-Yahr scale (mean±s.d.) | NA | NA | 2.0±0.4 | NA |
| UPDRS motor/bradykinesia subscale (off-medication; mean±s.d.) | NA | NA | 14.9±8.0/6.6±4.2 | NA |
| *LDE (mean±s.d.) | NA | NA | 554±218 | NA |

EHI: Edinburgh Handedness Inventory; MMSE: mini-mental status examination; “Experience” refers to personal history that may influence finger dexterity, such as typing, piano playing and so forth. Participants self-reported their experience in those activities in a simple yes-no questionnaire. UPDRS: Unified Parkinson’s Disease Rating Scale; ^†^: not significant with a two-sample *t*-test; ^‡^: not significant with a chi-square test. NA: not applicable
