# Supplementary figures and images for: A Common Function of Basal Ganglia-Cortical Circuits Subserving Speed in Both Motor and Cognitive Domains
Source: eNeuro. 2017 Dec 8;4(6):ENEURO.0200-17.2017. doi: 10.1523/ENEURO.0200-17.2017 (PMC5783269; doi:10.1523/ENEURO.0200-17.2017)

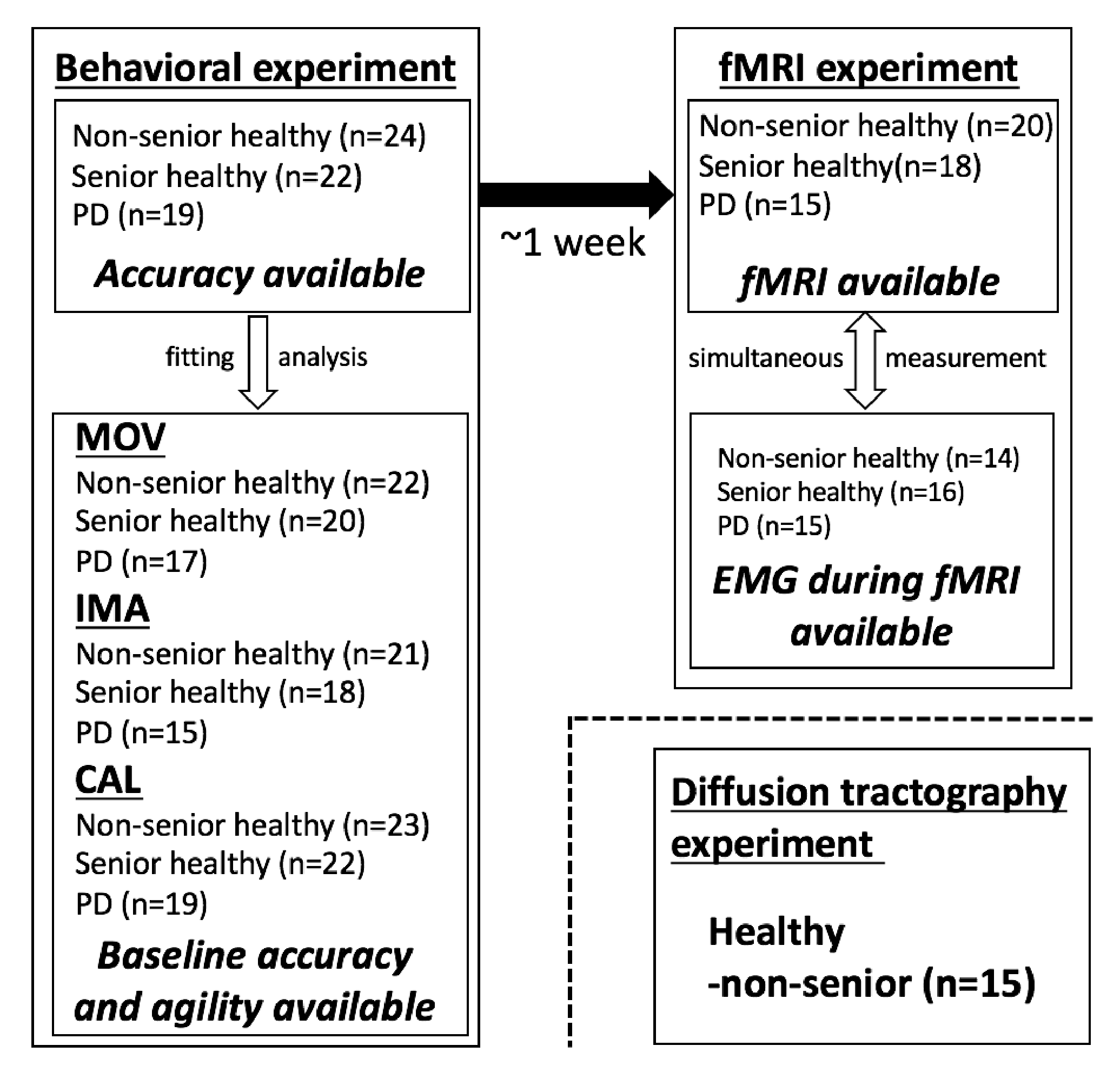

Supplement: Extended Data Figure 2-1 — Design of the experiment and data available for each analysis step. Forty-six healthy volunteers including 24 non-seniors and 22 seniors and 19 volunteers with mild to moderate PD participated in the behavioral experiment. Among those participants, 38 healthy volunteers (20 non-seniors and 18 seniors) and 15 volunteers with PD also participated in the fMRI experiment within one week after the behavioral experiment. After the fitting analysis of accuracy data in the behavioral experiment, Abase and Fmax parameters were available from 42 healthy (22 non-seniors and 20 senior) and 17 PD participants for the movement (MOV) task, 39 healthy (21 non-seniors and 18 seniors), and 15 PD participants for the imagery (IMA) task, and 45 healthy (23 non-seniors and 22 seniors) and 19 PD participants for the calculation (CAL) task. In the fMRI analysis, simultaneous EMG data were available from 30 healthy (14 non-seniors and 16 seniors) and 15 PD participants. We also ran a diffusion tractography experiment for diffusion-based subcortical classification in an independent group of 15 healthy non-senior participants. Download Figure 2-1, TIF file. [file sup_enu-eN-NWR-0200-17-s03.tif]

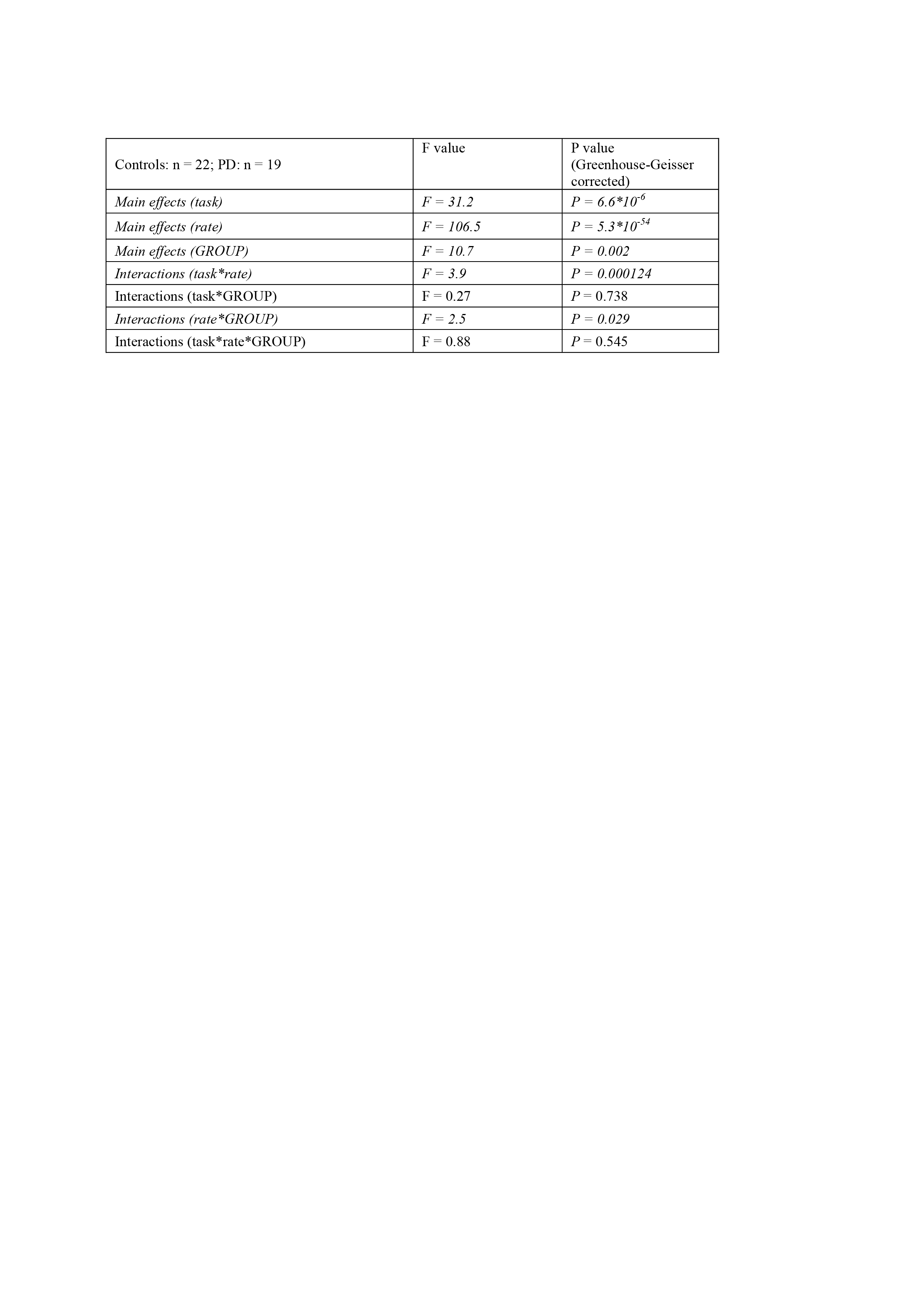

Supplement: Extended Data Figure 3-1 — Statistics from RM-ANOVA for the comparison between participants with PD (n = 19) and senior controls (n = 22) in the behavioral experiment. Significant results are shown in italic fonts. Download Figure 3-1, TIF file. [file sup_enu-eN-NWR-0200-17-s04.tif]

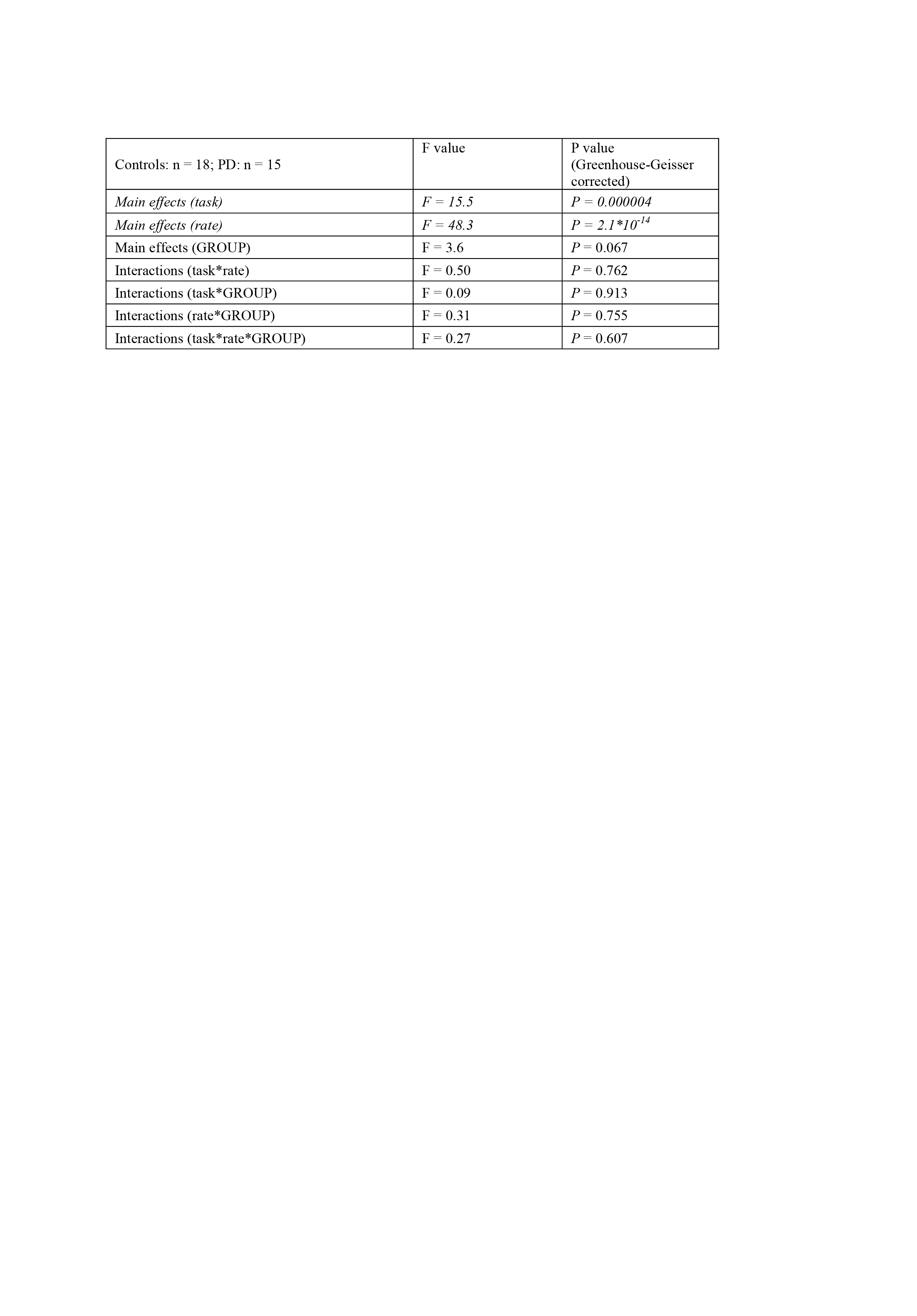

Supplement: Extended Data Figure 7-1 — Statistics from RM-ANOVA for the comparison between participants with PD (n = 15) and senior controls (n = 18) in the fMRI experiment. Significant results are shown in italic fonts. Download Figure 7-1, TIF file. [file sup_enu-eN-NWR-0200-17-s05.tif]
